# Rapid systematic review of readmissions costs after stroke

**DOI:** 10.1186/s12962-024-00518-3

**Published:** 2024-03-12

**Authors:** Pedro Abreu, Manuel Correia, Elsa Azevedo, Bernardo Sousa-Pinto, Rui Magalhães

**Affiliations:** 1 Department of Neurology, Centro Hospitalar Universitário de São João, Porto, Portugal; 2 Department of Clinical Neurosciences and Mental Health, Faculdade de Medicina, Universidade do Porto, Porto, Portugal; 3 Department of Neurology, Hospital Santo António– Centro Hospitalar Universitário de Santo António, Porto, Portugal; 4 Instituto de Ciências Biomédicas Abel Salazar, Universidade do Porto, Porto, Portugal; 5 MEDCIDS-Department of Community Medicine, Information and Health Decision Sciences, Faculdade de Medicina, Universidade do Porto, Porto, Portugal

**Keywords:** Stroke, Readmissions, Costs, Rapid review

## Abstract

**Background:**

Stroke readmissions are considered a marker of health quality and may pose a burden to healthcare systems. However, information on the costs of post-stroke readmissions has not been systematically reviewed.

**Objectives:**

To systematically review information about the costs of hospital readmissions of patients whose primary diagnosis in the index admission was a stroke.

**Methods:**

A rapid systematic review was performed on studies reporting post-stroke readmission costs in EMBASE, MEDLINE, and Web of Science up to June 2021. Relevant data were extracted and presented by readmission and stroke type. The original study’s currency values were converted to 2021 US dollars based on the purchasing power parity for gross domestic product. The reporting quality of each of the included studies was assessed using the Consolidated Health Economic Evaluation Reporting Standards (CHEERS) checklist.

**Results:**

Forty-four studies were identified. Considerable variability in readmission costs was observed among countries, readmissions, stroke types, and durations of the follow-up period. The UK and the USA were the countries reporting the highest readmission costs. In the first year of follow-up, stroke readmission costs accounted for 2.1–23.4%, of direct costs and 3.3–21% of total costs. Among the included studies, only one identified predictors of readmission costs.

**Conclusion:**

Our review showed great variability in readmission costs, mainly due to differences in study design, countries and health services, follow-up duration, and reported readmission data. The results of this study can be used to inform policymakers and healthcare providers about the burden of stroke readmissions. Future studies should not solely focus on improving data standardization but should also prioritize the identification of stroke readmission cost predictors.

**Supplementary Information:**

The online version contains supplementary material available at 10.1186/s12962-024-00518-3.

## Background

Stroke is a major worldwide health problem, with high mortality rates and a negative impact on the survivors’ quality of life [1, 2]. Stroke also incurs substantial health-related expenditures. In the European Union, its direct and indirect economic costs were estimated to reach $50 billion in 2015 [3]. In the United States of America (USA), these costs amounted to $73.7 billion in 2010 [4]. In the coming years, due to population aging and an increasing number of stroke survivors, the burden of this disease is expected to continue to rise [3]. In fact, the cost of stroke in the USA is estimated to reach up to $184.1 billion by 2030 [5]. 

Readmissions are one of the factors contributing to stroke-related costs. Hospital readmissions and frequent emergency service utilization following a first-ever stroke or transient ischemic attack (TIA) are common and increase stroke mortality and morbidity, thereby raising overall health costs [6, 7]. Readmissions may be associated, among others, with hospital quality of care, post-discharge transition services, and medical, social, and rehabilitation follow-up [6]. Despite some well-characterized limitations [8], readmissions are currently considered a measure of hospital performance and quality of care [4, 9, 10]. Given their impact on national health services’ expenditures [11], several countries (e.g., USA, United Kingdom (UK), and Germany) have implemented financial policies to reduce hospitalizations [12–14]. Nevertheless, the economic impact of stroke readmissions has not been systematically assessed. There is limited knowledge about the costs of post-stroke care, with the available systematic reviews not providing specific data on the costs of hospital readmissions [15]. 

Our rapid systematic review aims to provide information on the costs of hospital readmissions in patients with stroke as the primary diagnosis at index admission. We focused on readmission costs within the first year after stroke and its subtypes. Additional outcomes included readmission cost predictors, when stated.

## Methods

This rapid systematic review followed the recommendations of PRISMA Checklist [16]. 

### Search strategy and eligibility criteria

On June 26, 2021, we conducted a systematic literature search on the following bibliographic databases without applying time limits: EMBASE, MEDLINE (via Ovid and PubMed), and Web of Science. Appendix Supplemental Table 1 shows the full search strategy. We also manually screened the reference lists of included studies for additional citations.

We included primary studies that addressed readmission costs in patients aged 18 years and older with stroke as the primary diagnosis at index admission. The studies were longitudinal observational (retrospective and prospective) and experimental of any type, including articles with partial (e.g., cost-of-illness studies) or full economic evaluations (e.g., cost-effectiveness analysis).

We excluded studies that did not report readmission costs within the initial months or the first year following stroke (e.g., studies exclusively reporting costs between 4 and 12 months after stroke or focusing solely on costs during the second or third year after the index admission) and studies that exclusively examined particular stroke subtypes or specific stroke treatments/interventions (e.g., studies only addressing readmission costs in aneurismatic subarachnoid hemorrhage, atherosclerotic stroke, or thrombectomy). We also excluded studies in non-English or non-Romance languages.

### Study selection and data extraction

One of the authors conducted an initial eligibility screening based on titles/abstracts according to predetermined selection criteria. Three authors reviewed and discussed all studies that raised inclusion doubts, and disagreements were resolved by consensus. Then, one author reviewed the selected studies’ full text and included the ones that met the inclusion criteria in the final analysis. For articles excluded after full-text reading, reasons for exclusion were documented according to pre-specified criteria. One reviewer extracted all data relevant to our study: information on the author, country, publication year, stroke type, economic perspective, data sources on costs, healthcare and study setting, study design, period of study, follow-up period, control group (if applicable), population characteristics, sample size, type of estimation procedures, reported readmission costs (including currency reference year, cost data sources, cost estimation, overall costs, costs per readmission type, direct and indirect costs), readmission type (e.g., planned, unplanned, all-cause, stroke-recurrence or stroke-related complications), frequency of readmissions within the study period, readmission percentage of direct and total stroke costs and predictors of readmission costs.

Appendix supplementary table 2 describes the study approach classifications and definitions. Countries were divided into economic groups using the World Bank classification [17–22].

In cases where information regarding the average readmission cost or the percentage of direct or total stroke costs was not totally explicit, we made estimations by leveraging the data provided in the paper, whenever feasible (e.g., if a study only presented readmission costs for ischemic and hemorrhagic stroke, the weighted average of readmission stroke cost was calculated). The readmission costs were categorized as per index-hospitalization surviving patient, per patient, per patient alive at the end of study, per readmitted patient, and per readmission (Table 1). To standardize the included studies’ results, we converted their original currency values to 2021 US dollars (USD) using a web-based tool (CCEMG-EPPI-Centre cost converter v1.6) [23]. This tool converts reported costs into current-year costs of that country using the Gross Domestic Product Deflator Index (GDPI) followed by a conversion into USD using conversion rates based on the purchasing power parity for the gross domestic product (GDP) [23]. When the reported cost reference year was unavailable for estimating readmission costs, we considered the last year of the study period for this estimate.

**Table 1:** Readmission cost categories definitions

Readmissions cost categories	
Readmission cost per index-hospitalization surviving patient	Total cost of readmissions divided by the number of patients alive at the end of index-hospitalization
Readmission cost per patient	Total cost of readmissions divided by the number of study patients
Per-patient alive at the end of study	Total cost of readmissions divided by the number of patients alive at the end of study
Readmission cost per readmitted patient	Total cost of readmissions divided by the number of readmitted patients
Cost per readmission	Total cost of readmissions divided by the number of readmissions

### Data synthesis

We described the included studies’ results based on the total readmission costs and the costs per component and type of stroke. Given the variability of the included studies and the lack of comparability of readmission costs among different healthcare contexts and settings, we did not synthesize our findings using meta-analysis.

### Quality assessment of publications

One author assessed the reporting quality of the included studies using the Consolidated Health Economic Evaluation Reporting Standards (CHEERS) checklist [24]. Whenever the author was uncertain on how to assess the quality of a study in a certain item, such item was discussed and reviewed among three authors. Any disagreements were resolved through consensus.

## Results

### Search results and study description

**Fig. 1:**
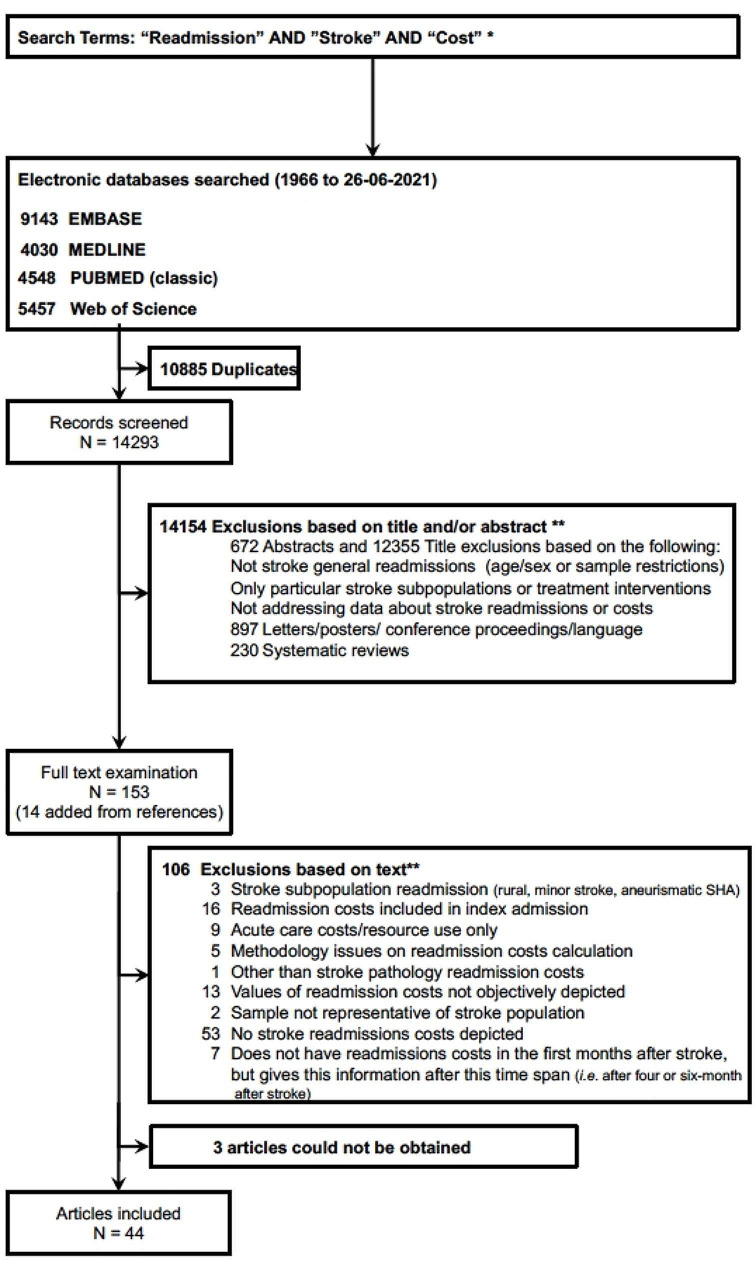
Flowchart of the studies’ identification and selection processes. *see Appendix Supplemental Table 1. ** Studies exclusion may have more than one reason. SHA- subarachnoid hemorrhage

The systematic literature search yielded 9143 references in EMBASE, 4030 in MEDLINE via Ovid, 4548 in MEDLINE via PubMed, and 5457 in Web of Science. After excluding 10,885 duplicates, 14,293 records were selected for title and abstract screening. This screening excluded 14,154 records. A total of 153 studies were selected for full evaluation, of which 14 were added after a manual review of the selected papers’ references. Three articles from the final list could not be obtained, and 106 studies were excluded. Thus, 44 articles were deemed suitable for inclusion in our systematic review (Fig. 1).

The included studies are described in Table 2 and Appendix Supplemental Tables 3 and 4. All studies were from high-income countries. The studies were conducted between 1975 [25] and 2015 [26], and the sample sizes ranged from 58 [27] to 985,851 participants [28]. The follow-up period ranged from seven days to 11 years and was most commonly between 6 and 12 months (26 studies). Most of the studies (34) reported readmission costs on all stroke subtypes, while others only addressed TIA (*N* = 2), ischemic stroke (IS) (*N* = 6), or intracerebral hemorrhage (ICH) (*N* = 1). One study that examined stroke readmission costs did not provide clear information regarding the specific stroke types included in their analysis. Most studies (*N* = 24) were prospective cohorts. Twenty publications assessed health expenditure (20 of 44) and cost of illness (20 of 44), while the remaining four encompassed one cost-minimization, one cost-benefit, and two cost-effectiveness analyses. Twenty-one studies adopted the limited-societal economic perspective, 20 addressed the healthcare payer and/or provider perspective, and three the healthcare sector perspective. Costs were estimated using a bottom-up approach in 26 studies, a top-down approach in 14 studies, and components of both types in four studies. The overall study costs were primarily obtained from questionnaires and/or hospital records (24 of 44), insurance administrative records (3 of 44), and regional (3 of 44) or national registers (14 of 44).

**Table 2:** Summary of the characteristics of the 44 studies included in this rapid review

Study characteristics	Number of studies	Reference(s)
**Research setting**		
United States of America	8	[25, 28, 30, 35, 49, 51, 67, 68]
Sweden	8	[27, 41, 42, 50, 54, 59–61]
Australia	5	[52, 55, 57, 58, 62]
Canada	4	[29, 37, 46, 53]
France, Germany, Italy, and United Kingdom	3	[26, 31, 32, 34, 36, 38, 43, 45, 56, 63, 65, 66]
Denmark and Taiwan	2	[33, 40, 47, 64]
Finland, Norway, and Spain	1	[39, 44, 48]
**Follow-up period**		
Up to 3 months	5	[28, 29, 53, 67, 68]
6–12 months	26	[25–27, 30–34, 38–47, 49, 51, 52, 54, 56–58, 64]
> 12 months	13	[35–37, 48, 50, 55, 59–63, 65, 66]
**Stroke type**		
All-stroke types	34	[25–27, 29, 31, 32, 34–36, 38–48, 50–63]
Ischemic	6	[30, 37, 64–67]
Intracerebral hemorrhage	1	[68]
Transient ischemic attack	2	[28, 33]
All-stroke types included not clear	1	[49]
First-ever	30	[25, 27–30, 33–36, 40, 45–53, 55–64, 68]
First-ever and recurrent	11	[26, 31, 32, 37–39, 43, 54, 65–67]
Not reported if first-ever or recurrent	3	[41, 42, 44]
**Study design**		
Prospective	24	[25, 26, 31–34, 38, 39, 43, 45, 46, 49, 50, 54, 55, 57–62, 64–66]
Retrospective	13	[28–30, 35–37, 40, 47, 51, 56, 63, 67, 68]
Both prospective and retrospective	1	[63]
Randomized trials	6	[27, 41, 42, 44, 52, 53]
**Calculation method**		
Health expenditures	20	[26, 28–30, 35–37, 39, 44, 47, 49, 51, 54, 56, 59, 63, 65–68]
Cost of illness	20	[25, 27, 31–34, 38, 42, 43, 45, 46, 48, 50, 55, 57, 58, 60–62, 64]
Cost minimization	1	[41]
Cost benefit	1	[53]
Cost effectiveness	2	[40, 52]
**Economic perspective**		
Limited societal	21	[25, 27, 31–34, 38, 41–43, 45, 46, 50, 52, 55, 57, 58, 60–62, 64]
Healthcare payer and/or provider	20	[26, 28–30, 35–37, 39, 40, 44, 47, 49, 51, 53, 54, 56, 59, 63, 67, 68]
Healthcare sector perspective	3	[48, 65, 66]
**Economic estimation**		
Bottom-up	26	[26, 27, 32–34, 38–43, 45–50, 52, 54, 55, 57, 58, 60–62, 64]
Top-down	14	[25, 28–30, 35–37, 44, 51, 56, 59, 63, 67, 68]
Both bottom-up and top-down	4	[31, 53, 65, 66]
**Economic data sources**		
Questionnaires and/or hospital records	24	[27, 32–34, 38, 39, 41–46, 49, 50, 52–55, 57, 58, 64–67]
Based only in insurance administrative data	3	[25, 26, 30]
Regional registries	3	[29, 37, 63]
National registries	14	[28, 31, 35, 36, 40, 47, 48, 51, 56, 59–62, 68]

### Quality appraisal

Appendix Supplemental table 5 shows a summary of the reporting quality appraisal. Most studies provided sufficient information on 23 of the 24 items listed in the CHEERS checklist [24] however, only 12 of 44 studies included the study discount rate or a conflict-of-interest statement, and 18 of 44 studies informed about the effects of uncertainty.

### Stroke readmission rates characterization

Readmission rates varied across studies, stroke types, and readmission types and were measured at different time points (Appendix Supplemental Table 4). The 30-day all-cause readmission rate ranged from 8.6% [29] to 39.0%; [26] the 1-year all-cause readmission rate ranged from 9.0% [31] to 50.5%; [26] the 30-day stroke-related readmission rate ranged from 3.2% [32] to 28.0%; [30] and the 1-year stroke-related readmission rate ranged from 7.8% [33] to 18.0% [33]. Two studies [35, 36] reported all-cause mean readmission rates of 56.4% and 60.7% at 11 years and 4 years of follow-up, respectively, while one study [37] reported a mean stroke-related readmission rate of 72.7% at 5 years.

### Readmission cost characterization

Appendix Supplemental table 4 shows a global description of stroke readmission costs. Out of the 44 studies, 35 addressed stroke readmission costs of all-subtypes (table 2). Of these 35 studies, 23 addressed the costs of readmissions due to all causes, [26, 27, 29, 31, 35, 36, 38–54] and 13 due to stroke recurrence or its complications, [25, 32, 34, 39, 55–63] eight of these studies (8/35) also included TIA readmission costs [26, 31, 34, 38, 40, 46, 47, 63.

Table 3 presents the readmission costs by geographic region for all-cause readmissions and readmissions related to stroke recurrence and/or complications irrespective of the follow-up period. The UK ($11,651) and the USA ($9722) exhibited the highest mean readmission costs, while Australia ($905) had the lowest mean readmission costs.

**Table 3:** Stroke readmission costs by geographic region. *

Geographic Region	n	Mean, $	Median, $	Range	References
Sweden	7	3639	1860	400 − 12,081	[41, 42, 50, 54, 59–61]
UK	3	11,651	5604	1203-28,417	[36, 56, 63]
Rest of Europe	9	1892	1274	104–6099	[31, 32, 34, 38, 39, 43–45, 48]
Australia	4	905	828	120–1404	[55, 57, 58, 62]
Taiwan	2	3545	3545	-	[40, 47]
Canada	3	3630	1649	650 − 10,574	[29, 46, 53]
USA	4	9722	8836	4017-17,198	[25, 35, 49, 51]

UK, United Kingdom; USA, United States of America

*Includes readmission costs for all-cause readmissions and readmissions related to stroke recurrence and/or complications, encompassing different readmission categories such as per surviving patient, per patient, or per surviving patient at the end of the study, irrespective of the follow-up duration. Also, whenever the same study gave various readmission costs categories only the per surviving patient readmission cost was accounted for

Table 4 presents the stroke readmission costs based on readmission subtypes and the length of follow-up. Most studies (*N =* 24) [26, 27, 31, 34, 38–44, 46, 47, 49, 50, 52, 54, 56–62] reported costs within the 12-month follow-up period. In addition, 21 studies [29, 31, 32, 36, 38, 39, 41–45, 49, 51, 53–58, 62] presented costs per index-hospitalization surviving patient and ten studies [35, 36, 40, 46–48, 50, 56, 59, 61] per patient.

**Table 4:** Stroke readmission costs based on readmission subtypes and length of follow-up

All-cause readmissionsª					
	Per-index hospitalization surviving patient				
Follow-up	n	Mean, $	Median, $	Range, $	References
≤ 3-month	2	1316	1372	650–1925	[29, 53]
> 3 - ≤ 6-month	2	2274	2274	531–4017	[45, 51]
12-month	9	3677	2127	755 − 13,538	[31, 38, 39, 41–44, 49, 54]
> 12-month	1	28,417	28,417	-	[36]
	Per-patient				
Follow-up	n	Mean, $	Median, $	Range, $	References
≤ 3-month	-	-	-	-	-
> 3 - ≤ 6-month	-	-	-	-	-
12-month	5	4965	3545	1063-10,574	[40, 46–48, 50]
> 12-month	2	18,393	18,393	17,198 − 19,588	[35, 36]
	Per-readmitted patient				
Follow-up	n	Mean, $	Median, $	Range, $	References
≤ 3-month	1	6019	6109	-	[52]
> 3 - ≤ 6-month	-	-	-	-	-
12-month	1	6664	6664	-	[52]
> 12-month	-	-	-	-	-
	Per-readmission				
Follow-up	n	Mean, $	Median, $	Range, $	References
≤ 3-month	1	10,472	10,472	8083-12,861	[29]
> 3 - ≤ 6-month^++^	1	-	983	0-4375	[26]
12-month	2	10,464	10,464	2956-17,971	[27, 49]
> 12-month	1	7316	7316	-	[36]
	Per-patient alive at the end of study				
Follow-up	n	Mean, $	Median, $	Range, $	References
≤ 3-month	-	-	-	-	-
> 3 - ≤ 6-month^++^	-	-	-	-	-
12-month	1	3035	3035	-	[41]
> 12-month	-	-	-	-	-
Stroke-recurrence and/or related/complications readmissions*					
	Per-index hospitalization surviving patient				
Follow-up	n	Mean, $	Median, $	Range, $	References
≤ 3-month	2	720	720	236–1203	[32, 63]
> 3 - ≤ 6-month	-	-	-	-	-
12-month	4	1842	993	104–6962	[39, 56–58]
> 12-month	2	784	828	120–1404	[55, 62]
	Per-patient				
Follow-up	n	Mean, $	Median, $	Range, $	References
≤ 3-month	-	-	-	-	-
> 3 - ≤ 6-month	-	-	-	-	-
12-month	3	4920	3599	400 − 12,081	[56, 59, 61]
> 12-month	-	-	-	-	-
	Per-readmitted patient				
Follow-up	n	Mean, $	Median, $	Range, $	References
≤ 3-month	-	-	-	-	-
> 3 - ≤ 6-month		-	-	-	-
12-month	1	15,183	15,183	-	[56]
> 12-month					
	Per-readmission				
Follow-up	n	Mean, $	Median, $	Range, $	References
≤ 3-month	-	-	-	-	-
> 3 - ≤ 6-month	-	-	-	-	-
12-month	2	17,592	17,592	8396-26,788	[56, 62]
> 12-month	-	-	-	-	-
	Per first-stroke survivor				
Follow-up	n	Mean, $	Median, $	Range, $	References
≤ 3-month	-	-	-	-	-
> 3 - ≤ 6-month	-	-	-	-	-
12-month	1	4143	4143	-	[25]
> 12-month	-	-	-	-	-
	Per-patient alive at the end of study				
Follow-up	n	Mean, $	Median, $	Range, $	References
≤ 3-month	-	-	-	-	-
> 3 - ≤ 6-month	-	-	-	-	-
12-month	2	1515	1515	1399–1630	[34, 61]
> 12-month	-	-	-	-	-

ª Some study author´s calculated different costs for all-cause readmissions

*ªSome study author´s calculated different costs for stroke-recurrence and/or stroke complications

^++^Deutschbein et al. only calculated the 6-month median per readmission (standard care group $983 (range: $0-$4,375) and intervention group $0 (range: $0-$2,726))

The mean all-cause readmission cost per index-hospitalization surviving patient and per patient in the 12-month follow-up period was $3677 and $4965, respectively. In these readmission categories, during this follow-up period, the lowest mean readmission costs were observed in Italy [43] ($755) and Spain [39] ($969), while the highest were reported in the USA [49] ($13,538). Overall, the highest annual all-cause readmission costs per index-hospitalization surviving patient and per patient were reported in studies with longer follow-up periods, respectively, in Scotland by McGuire et al. [36]($21,417) and in the USA by Lee et al. [35] ($17,198).

In the 12-month follow-up period, the mean annual costs of readmission due to stroke recurrence and/or complications per index-hospitalization surviving patient and per patient were, respectively, $1842 and $4962. In these readmission types and period, the lowest mean readmission costs were observed in Spain ($104) [39] and the highest in Sweden ($12,081) [59]. Only two studies (from Australia) registered costs of readmission due to stroke recurrence and/or stroke-related complications per index-hospitalization surviving patient in a follow-up period > 12 months, reporting mean costs of $828 [55, 62]. 

**Fig. 2:**
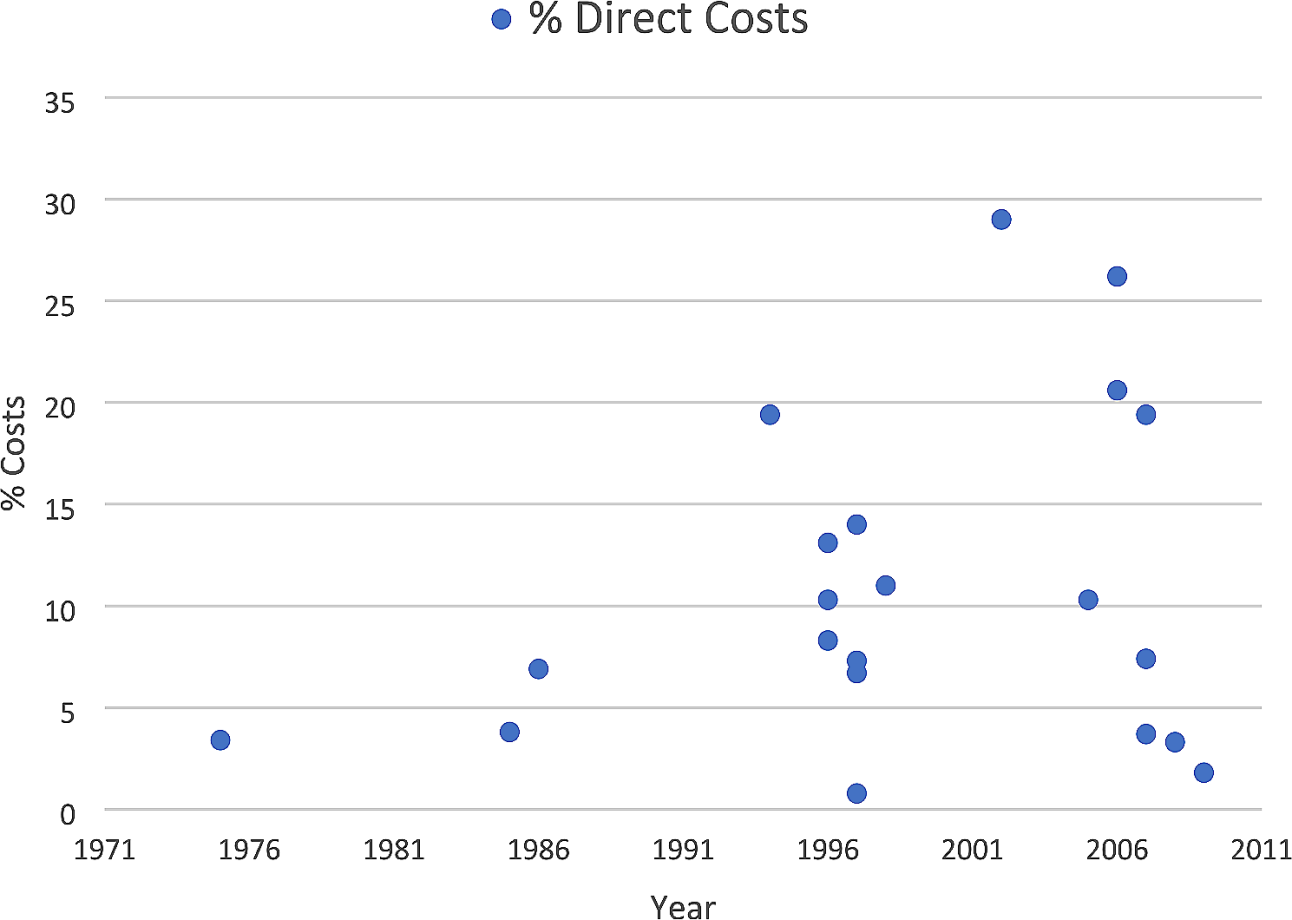
Proportion of readmission costs in relation to direct stroke costs (12-month follow-up period)

**Fig. 3:**
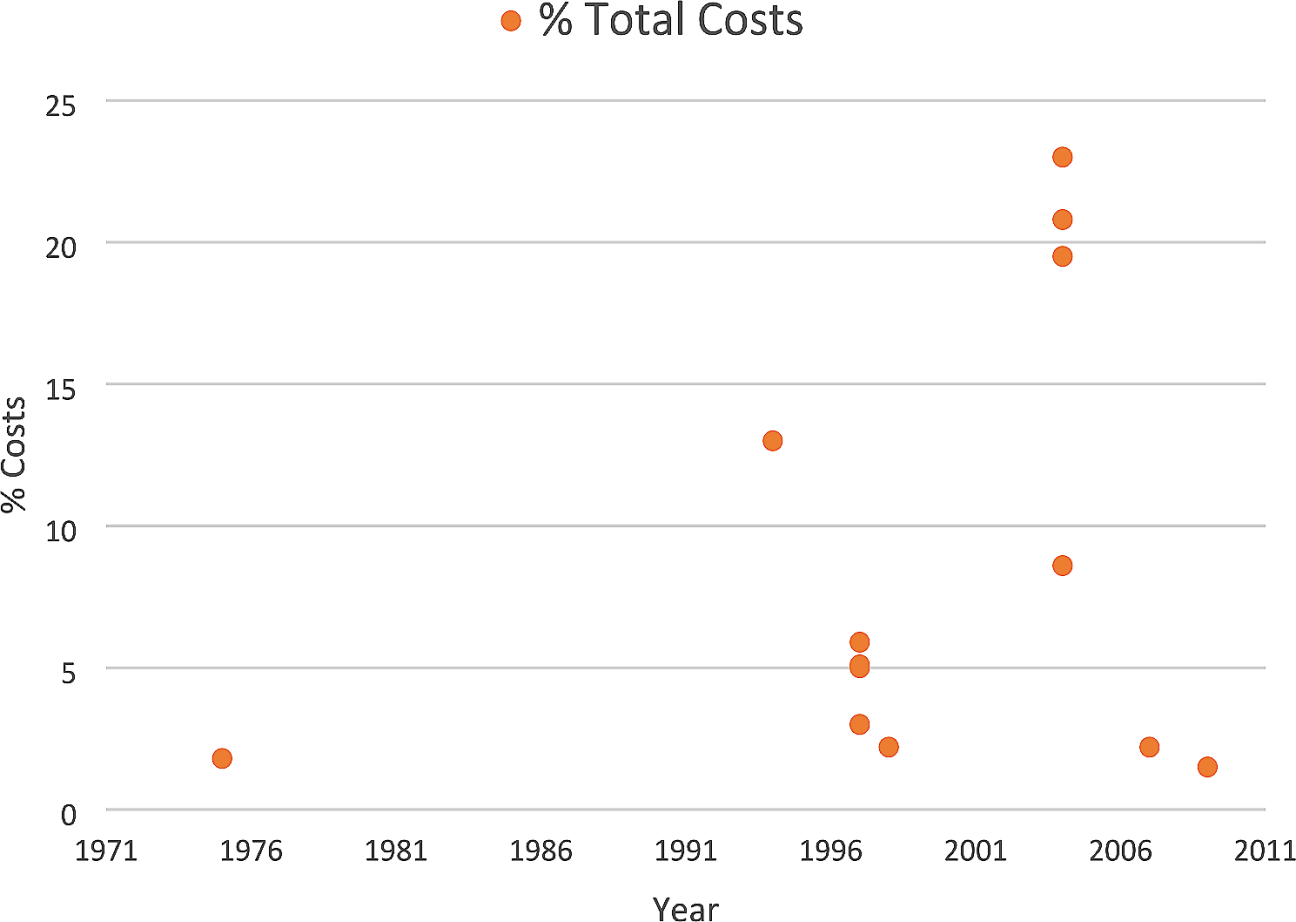
Proportion of readmission costs in relation to total stroke costs (12-month follow-up period)

In the first year of follow-up, the readmissions represented 0.78% [39] to 29.0% [40, 47] of direct and 1.5% [60] to 23.0% [46] of total stroke costs (Figs. 2 and 3), and these proportions tended to increase in more recent years.

### Readmission cost by stroke type

#### Transient ischemic accident

Five of the reviewed studies provided data on readmission costs in TIA (Appendix Supplemental Table 6) [28, 33, 46, 47, 63]. In a 12-month follow-up, the mean annual all-cause readmission cost was reported per index-hospitalization surviving patient in one study from Denmark [33] ($360) and per patient in two studies, from Canada [46] ($9161) and Taiwan [47] ($3037). Across the studies, TIA readmission costs represented 6.2% [47] to 29.8% [63] of TIA direct costs and 46% [46] of TIA total costs.

#### Ischemic stroke

We identified data on IS readmission costs in 16 studies (Appendix Supplemental Table 7) [29, 30, 35–37, 40, 46–48, 55, 56, 62, 64–67]. Table 5 depicts the stroke readmission costs by readmission subtypes and length of follow-up.

**Table 5:** Ischemic stroke readmission costs based on readmission subtypes and length of follow-up

All-cause readmissions					
	Per-index hospitalization surviving patient				
Follow-up	n	Mean, $	Median, $	Range	References
≤ 3-month	1	1284	1284	642–1925	[29]
> 3 - ≤ 6-month	-	-	-	-	-
12-month	1	1010	1010	-	[66]
> 12-month	2	14,903	14,903	1166-28,640	[36, 65]
	Per-patient				
Follow-up	n	Mean, $	Median, $	Range, $	References
≤ 3-month	1	54,212	54,212	-	[67]
> 3 - ≤ 6-month	-	-	-	-	
12-month	3	7629	6486	3768-12,632	[46–48]
> 12-month	2	18,736	18,736	17,453 − 20,019	[35, 36]
	Per-readmission				
Follow-up	n	Mean, $	Median, $	Range, $	References
≤ 3-month	1	10,065	10,065	7729-12,401	[29]
> 3 - ≤ 6-month	-	-	-	-	-
12-month	-	-	-	-	-
> 12-month	1	7308	7308	-	[36]
Stroke-recurrence and/or related/complications readmissions**					
	Per-index hospitalization surviving patient				
Follow-up	n	Mean, $	Median, $	Range, $	References
≤ 3-month	-	-	-	-	-
> 3 - ≤ 6-month	-	-	-	-	-
12-month	2	4530	4530	2043–7016	[56, 64]
> 12-month	2	844	857	132–1542	[55, 62]
	Per-patient				
Follow-up	n	Mean, $	Median, $	Range, $	References
≤ 3-month	-	-	-	-	-
> 3 - ≤ 6-month	-	-	-	-	-
12-month	1	5926	5926	-	[56]
> 12-month	-	-	-	-	-
	Per-readmitted patient				
Follow-up	n	Mean, $	Median, $	Range, $	References
≤ 3-month	-	-	-	-	-
> 3 - ≤ 6-month	-	-	-	-	-
12-month	1	15,672	15,672	-	[56]
> 12-month	-	-	-	-	
	Per-readmission				
Follow-up	n	Mean, $	Median, $	Range, $	References
≤ 3-month	1	13,807	13,807	-	[30]
> 3 - ≤ 6-month	-	-	-	-	-
12-month	1	17,646	17,646	8504-26,788	[56, 62]
> 12-month	-	-	-	-	-

**Some study author´s calculated different costs for stroke-recurrence and/or stroke complications

At 12 months of follow-up, the mean annual all-cause readmission costs per index-hospitalization surviving patient and per patient were, respectively, $1010 and $7629. The lowest mean readmission costs were observed in France ($1010) [66] and the highest in Canada ($12,632) [46]. In this period, the mean annual costs of readmission due to stroke recurrence and/or stroke-related complications per index-hospitalization surviving patient and per patient were, respectively, $4350 and $5926. In this readmission category, the lowest mean readmission costs were observed in Denmark [33] ($2043) and the highest in Scotland [56] ($7016).

We observed that the highest annual readmission costs tended to occur in studies with longer follow-up periods, with a notable exception of the study conducted by Stein et al. (USA) [67], which registered a 30-day all-cause readmission cost per patient of $54,212. Moreover, in the first year of follow-up, readmission costs accounted for 2.1% [62] to 23.4% [48], of direct costs and 3.3% [66] to 21.0% [46] of total costs.

### Intracerebral hemorrhage

Data on readmission costs of ICH were identified in 11 studies (Table 6 and Appendix Supplemental Table 8) [29, 35, 36, 40, 45–48, 55, 56, 62, 68], although one of these studies provided readmission costs only for both intracerebral and subarachnoid hemorrhage (SAH) [47]. In a 12-month follow-up period, the all-cause intracerebral hemorrhage readmission costs per patient were reported by Lee et al. [47] in Taiwan ($4102), Meretoja et al. [48] in Finland ($5009), and Goere et al. [46] in Canada ($5905). Only one study, conducted by Christensen et al. [56] (Scotland), described the 12-month stroke-recurrence readmission costs per patient surviving an ICH index hospitalization ($6624) and per patient ($3635). In the aforementioned readmission categories, the 12-month readmission ICH percent of direct costs ranged from 2.8% [29] to 35.6% [47], and the readmission percent of total ICH costs was of 9.0% [46].

**Table 6:** Hemorrhagic stroke readmission costs based on readmission subtypes and length of follow-up

All-cause readmissions					
	Per-index hospitalization surviving patient				
Follow-up	n	Mean, $	Median, $	Range	References
≤ 3-month	1	1240	1240	679–1801	[29]
> 3 - ≤ 6-month	-	-	-	-	-
12-month		-	-	-	-
> 12-month	1	26,041	26,041	-	[36]
	Per-patient				
Follow-up	n	Mean, $	Median, $	Range, $	References
≤ 3-month	1	54,212	54,212	-	[67]
> 3 - ≤ 6-month	-	-	-	-	
12-month	3	4609	4102	3821–5905	[46–48]
> 12-month	2	15,585	15,585	14,147 − 17,022	[35, 36]
	Per-readmission				
Follow-up	n	Mean, $	Median, $	Range, $	References
≤ 3-month	2	11,677	10,777	679 − 14,732	[29, 68]
> 3 - ≤ 6-month	-	-	-	-	-
12-month	-	-	-	-	-
> 12-month	1	6321	6321	-	[36]
Stroke-recurrence and/or related/complications readmissions**					
	Per-index hospitalization surviving patient				
Follow-up	n	Mean, $	Median, $	Range, $	References
≤ 3-month	-	-	-	-	-
> 3 - ≤ 6-month	-	-	-	-	-
12-month	1	6624	6624	-	[56]
> 12-month**	2	347	492	32–516	[55, 62]
	Per-patient				
Follow-up	n	Mean, $	Median, $	Range, $	References
≤ 3-month	-	-	-	-	-
> 3 - ≤ 6-month	-	-	-	-	-
12-month	1	3635	3635	-	[56]
> 12-month	-	-	-	-	-
	Per-readmitted patient				
Follow-up	n	Mean, $	Median, $	Range, $	References
≤ 3-month	-	-	-	-	-
> 3 - ≤ 6-month	-	-	-	-	-
12-month	1	15,183	15,183	-	[56]
> 12-month	-	-	-	-	-
	Per-readmission				
Follow-up	n	Mean, $	Median, $	Range, $	References
≤ 3-month	1	13,807	13,807	-	[30]
> 3 - ≤ 6-month	-	-	-	-	-
12-month	2	17,206	17,206	7623-26,788	[56, 62]
> 12-month	-	-	-	-	-

**Some study author´s calculated different costs for stroke-recurrence and/or stroke complications

### Subarachnoid hemorrhage

The readmission costs of SAH were reported in three of the 44 studies (Appendix Supplemental Table 9) [35, 47, 48]. These studies were conducted in Taiwan [47], Finland [48], and the USA [35], estimating all-cause mean annual per-patient readmission costs of $945, $5009, and $21,342, respectively. Readmission costs accounted from 12.5 [48] to 31.9% [35] of direct readmission costs.

### Readmission cost predictors

Of the 44 reviewed studies, six focused on readmission costs as their primary study objective, while the remaining studies provided a global assessment of stroke or post-stroke care costs [28, 37, 47, 56, 67, 68]. Stein et al. [67] was the only one that identified readmission after IS in another hospital as a predictor of increased readmission charges.

## Discussion

To the best of our knowledge, this study is the first systematic attempt to analyze readmission costs in stroke patients. We provide detailed data on stroke readmission costs from 44 publications, adjusted to 2021 US dollars. We observed considerable variability across studies regarding study design, follow-up period, and reported readmission data. Moreover, differences in readmission costs were observed both across different countries and within the same country, with the UK and the USA reporting the highest annual readmission costs. We also found that the impact of readmissions on direct and total stroke costs varied across studies. Only one study identified predictors of readmission costs.

The readmissions occurring up to one year after a stroke hospitalization are important as they are associated with both preventable and non-preventable factors that can predict readmissions [69, 70]. Most of the included studies reported readmission rates and costs within this follow-up period. We observed a substantial variation in readmission rates, not only at the one-year mark but also within the 30-day period. Such a substantial disparity can be attributed to the diverse study methodologies employed and the distinct characteristics of national healthcare systems [71]. Also, and as anticipated, studies with longer follow-up times showed a higher percentage of patients with readmissions [35–37]. However, even in studies assessing patients for several years, readmissions were more likely to occur in the first years after the index stroke, especially within the first year [35–37]. This suggests a relatively acute effect of the index stroke hospitalization in the readmission rates [37]. Consequently, readmission costs would be expected to be higher in the first months or years after the index stroke, as observed by Ghatnekar et al. [59, 60] and Caro et al [37]. Nevertheless, caution should be exercised in attributing all readmissions, and consequently, all associated costs, to the index stroke, as many would occur because of the patient’s age and comorbidities [37]. 

All readmission costs were presented in 2021 USD [23], adjusted for variations in relative prices between economies, as recommended for cross-country cost comparisons [15, 72, 73]. However, even after accounting for these adjustments, significant discrepancies in readmission costs were identified across different countries and - within the same country - across readmission categories [36, 41, 50, 51, 56]. In the analysis of stroke readmission costs higher costs were frequently observed in the UK, namely Scotland, and the USA. Notably, these countries were also the ones where studies with longer follow-up periods were conducted, thus reporting the highest readmission costs. Conversely, Spain and Australia exhibited the lowest costs in our analysis. Besides methodological differences, these variations in readmission costs may reflect disparities in stroke care organization and the studies’ temporal context (potentially reflecting rising healthcare expenditures over time in developed countries) or diverse healthcare models.

We perceived that most of the studies provided readmission cost data considering all-cause readmission, and fewer had information about stroke recurrence or stroke-related/complications readmission costs. On one hand, to better account for the impact of stroke on readmission costs it would be preferable to use a stroke-specific cause of readmission [74], on the other hand, it could be argued that studies only including the costs that were directly attributable to the event might not include all the costs associated with the disease [75]. In forthcoming works, if the objective is to capture the global costs of readmission after a stroke index hospitalization, it would be important, in theory, that both types of readmissions are registered.

The different reported categories of readmission costs as per index-hospitalization surviving patient, per patient, per surviving patient, per patient readmitted and per readmission were another important source of heterogeneity identified. This aspect may also explain the rationale behind encountering relatively low readmission costs in some cases. Most of the studies captured readmission costs only as part of their total stroke or post-stroke costs estimates. Consequently, the method of calculating readmission costs in these studies diverged based on their primary objectives. For instance, those studies focusing on the post-stroke costs or randomized studies tended to calculate their costs as per index-hospitalization surviving patient [37, 44, 52]. Nonetheless, to better address this matter, it would be advisable that future studies would consider presenting calculations of stroke readmission costs covering all the aforementioned categories, as exemplified by the approach taken by Christensen et al [56].

Regarding readmission costs per stroke subtype, most studies focused on IS and ICH. The highest mean costs of all-cause readmissions per patient and per index-stroke-surviving patient in the 12-months follow-up were observed in SAH and IS, respectively. Additionally, the highest stroke-recurrence or stroke-related readmission costs were generally observed in IS (Table 5 and Appendix Supplemental Table 7). These findings may result from a relative scarcity of studies examining readmission costs specifically related to SAH. Additionally, this might reflect a survivorship bias, as IS is associated with higher survival rates following an index stroke, and patients who died during the index admission are not eligible for readmission analysis [48, 66, 68]. 

Overall, readmissions accounted for a wide range of direct (0.78% [39] to 53.5% [36]) and total stroke costs (1.5% [60] to 46% [46]). This variation may reflect differences in follow-up periods across studies. However, even when considering the 12-month period, a significant disparity in estimates persisted. Another potential factor contributing to this disparity is the inclusion of different cost data. For example, some studies [62], [46] incorporated costs related to informal care, out-of-pocket expenses, and productivity loss in their resource categories, while others [36] only considered hospitalization costs. Additionally, few studies provided information on non-medical costs associated with readmissions, possibly because many studies were not specifically designed to examine readmission costs, often considering them as a sub-category within the broader context of total stroke costs. Furthermore, distinguishing between direct and indirect consequences of disease and treatment costs can be challenging, as different consequences may be classified as “direct” costs depending on the analysis perspective [19]. To address these gaps in the existing literature, we suggest that future studies investigating readmission costs thoroughly examine the specific impact of readmissions considering both medical and non-medical cost. Moreover, our study observed that, in the more recent years, readmissions tended to be responsible for a higher proportion of stroke direct costs, which could be attributed to rising healthcare costs over time. Changes in healthcare policies and reimbursement systems may also impact the financial aspects of readmissions. Further research should consider these factors for a comprehensive understanding of the underlying reasons for the observed increase in direct readmission costs.

Stein et al. [67] demonstrated an association between readmission to a different hospital and increased total readmission charges, emphasizing the need for a good transition of care and follow-up after a stroke. Although none of the remaining studies assessed predictors of readmission costs, several of them identified predictors associated with increased readmission rates in stroke survivors and potentially indirectly associated with higher readmission costs. The recognition of these potentially modifiable predictors is important since it may help healthcare organizations better allocate resources and implement targeted readmission reduction policies [71, 76]. 

The strength of this rapid systematic review is that we conducted a thorough search and considered not only overall stroke readmissions costs but also costs per component and per type of stroke. Moreover, it assessed the impact of readmission costs on direct and total stroke costs. As in another studies [8, 77] our work also identifies several knowledge gaps about post-stroke readmission costs and suggests several aspects that can be improved in future works (Table 7).

**Table 7:** Overview of methodological aspects of stroke readmission cost and improvement suggestions

	Methodological aspects	Problem	Improvement suggestions
**Time window**	Different time windows for readmission cost calculation.	Discrepancies in readmission costs were identified among different time frames.	Evaluate and give readmission cost in different time windows (≤ 3-month, 6 month, 12-month, > 12 month) whenever possible [79] or give a readmission cost weighted average per month [15]
**Readmission Type**	Different readmission types were used for readmission cost calculations.	This variability makes it difficult to determine the true impact of stroke in readmission costs.	Report data on costs associated with both types of readmissions (all-cause and cause-specific).
**Effect of competing variables**	Diverse readmission categories with or without competing variables exclusion, such as mortality, were observed.	Not excluding the patients who died during their index stroke hospitalization, may underestimate the readmission cost.	To understand the diversity of stroke readmission costs it would be advisable to report readmission cost data using all the readmission categories (e.g., per patient, per surviving patient, per surviving patient at the study end).
**Different cost data**	Inclusion of diverse cost data.	Readmissions accounted for a wide range of direct and total stroke costs. Few studies provided information on non-medical costs.	Studies investigating readmission costs should thoroughly examine the specific impact of readmissions considering both medical and non-medical cost.
**Clinical setting and healthcare policies**	Readmission may reflect the clinical setting where stroke patients are treated and different healthcare policies.	Specialized stroke unities and care after discharge may influence readmissions [8].	Readmission costs should be linked to the diverse clinical setting and the countries healthcare policies [8, 15].
**Readmission cost predictors**	Paucity of readmission cost predictors data.	The readmission cost predictors are underreported.	To better allocate healthcare resources future research should include readmission cost predictors.

Our review has some limitations. Firstly, the studies were retrieved and assessed by a single reviewer. Secondly, publication bias may have occurred since we limited our search to studies published in English or Romance languages. Thirdly, the wide variations in the studies’ definitions of perspectives and types of readmission costs may have caused information biases.

Furthermore, all of the included studies were conducted in countries classified as high-income by the World Bank [20]. This means that our findings may not be generalizable to low and middle-income countries, where post-stroke care costs may be lower [15]. 

As in similar works [15, 75, 78], our review’s comparison of costs data was hampered by considerable methodological and clinical variability among studies. This variation made it difficult to compare readmission costs across different countries and within the same country. Furthermore, the included studies reported costs on different economic units, and discount rates were not always reported. Likewise, the definition of direct or indirect readmission costs may have varied across the studies, potentially leading to an inaccurate representation of their true financial burden. However, we used a web-based tool [23] to adjust for relative price differences between economies and to better compare costs across countries.

We did not study acute stroke care supply and practice patterns in each country, which may have influenced readmission costs. For instance, some of the included studies were carried out before the implementation of stroke units and the more recent recanalization therapies, which may further explain the observed variability of readmission rates and their costs.

Our study did not utilize the annual GDP to compare stroke readmission costs across countries. Including GDP data in cost comparisons would have allowed for a deeper understanding of the economic context and relative affordability of readmission costs in different nations. Consequently, further research in this field is warranted to address this knowledge gap.

The methodological limitations of this review have also been identified in other studies that performed international comparisons and faced difficulties in comparing studies based on different countries and costs [15, 72, 78]. We agree with Rajsic et al [15] that there is a need to create a methodological and clinically standardized supported list of segments of services that should be considered when reporting the cost of care.

## Conclusion

Our rapid systematic review represents the first comprehensive assessment of stroke readmission costs. We synthesized the readmission cost data from 44 studies conducted in 13 countries. Our findings emphasize the substantial financial burden of readmissions on the health care institutions and on patients, their families, and caregivers. Future research should focus on improving data standardization and expand efforts to include not only health-related expenditures but also the predictors and non-health consequences of stroke readmissions.

### Electronic supplementary material

Below is the link to the electronic supplementary material.

Supplementary Material 1

Supplementary Material 2

Supplementary Material 3

Supplementary Material 4

Supplementary Material 5

Supplementary Material 6

Supplementary Material 7

Supplementary Material 8

Supplementary Material 9

## Data Availability

The datasets used and/or analyzed during the current study are available from the corresponding author on reasonable request.

## Abbreviations

ICH: Intracerebral hemorrhage
IS: Ischemic stroke
GDP: Gross domestic product
SHA: Subarachnoid hemorrhage
TIA: Transient ischemic attack
USA: United States of America
USD: US dollars
UK: United Kingdom
