# Supplementary material for: Rapid systematic review of readmissions costs after stroke
Source: Cost Eff Resour Alloc. 2024 Mar 12;22:22. doi: 10.1186/s12962-024-00518-3 (PMC10936094; doi:10.1186/s12962-024-00518-3)
Supplement: Supplementary file 1 — Supplementary Material 1 [file 12962_2024_518_MOESM1_ESM.pdf]

Appendix Supplemental Table 1 - Search strategies for the included databases

Embase®

Embase Session Results

No.	Query	Results
#28	#27 AND 'human'/de AND ([adult]/lim OR [aged]/lim OR [middle aged]/lim OR [very elderly]/lim OR [young adult]/lim)	9,143
#27	#5 AND #18 AND #26	16,534
#26	(#19 OR #20 OR #21 OR #22 OR #23 OR #24 OR #25) AND [1966-2021]/py AND [1-1-1966]/sd NOT [27-6-2021]/sd	2,736,519
#25	(value NEAR/5 (money OR monetary)):ti,ab	4,002
#24	((resource OR healthcare OR health*care) NEAR/5 (use OR utilization OR cost*)):ti,ab	95,439
#23	'health utilit*':ti,ab OR 'economic evaluation*':ti,ab OR 'economic review*':ti,ab OR 'economic model*':ti,ab OR 'budget* impact analys*s':ti,ab OR burden:ti,ab	393,879
#22	(charge* NEAR/5 hospital):ti,ab	8,108
#21	(cost* NEAR/2 (effective* OR utilit* OR benefit* OR minimi* OR analy* OR effective* OR outcome OR outcomes OR illness OR measure OR comparison OR estimate* OR hospital OR 'long term')):ti,ab	299,767
#20	economic*:ti,ab OR cost*:ti,ab OR price*:ti,ab OR pricing:ti,ab OR pharmaco*economic*:ti,ab OR expend*:ti,ab OR expens*:ti,ab OR financ*:ti,ab OR resource*:ti,ab OR reimburs*:ti,ab OR budget*:ti,ab	1,917,882
#19	'economics'/exp OR 'costs and cost analysis'/exp OR 'economics, nursing'/exp OR 'economics, medical'/exp OR 'economics, pharmaceutical'/exp OR 'economics, hospital'/exp OR 'fees and charges'/exp OR 'budgets'/exp OR 'hospital costs'/exp OR 'health care costs'/exp OR 'health expenditures'/exp OR 'health resources'/exp OR 'cost of illness'/exp OR 'models, economic'/exp OR 'fee'/exp OR 'cost'/exp	1,220,471
#18	(#6 OR #7 OR #8 OR #9 OR #10 OR #11 OR #12 OR #13 OR #14 OR #15 OR #16 OR #17) AND [1966-2021]/py AND [1-1-1966]/sd NOT [27-6-2021]/sd	953,388
#17	hemiplegia:ti,ab OR hemiparesis:ti,ab OR aphasia:ti,ab OR dysphasia:ti,ab OR hemianopia:ti,ab	56,044
#16	((brain* OR cerebr* OR cerebell* OR intracran* OR intracerebral OR supratentorial OR infratentorial) NEAR/5 ('venous malformation' OR 'arteriovenous malformation')):ti,ab	2,826
#15	(('transient isch*mic*' OR 'isch*mic* reversible') NEAR/5 ('attack*' OR 'episode*' OR 'deficit*' OR 'stroke*' OR 'neurologic*' OR 'accident*')):ti,ab	24,745
#14	(thrombos* NEAR/5 (intracranial OR 'venous sinus' OR 'sagittal sinus' OR 'sagittal vein')):ti,ab	4,943
#13	((brain* OR cerebr* OR cerebell* OR intracran* OR intracerebral OR supratentorial OR infratentorial OR vertebrobasilar OR brainstem OR subarachnoid OR aneurism) NEAR/5 (haemorrhage* OR hemorrhage* OR hematoma* OR haematoma* OR bleed*)):ti,ab	112,713
#12	((carotid OR brain* OR cerebr* OR cerebell* OR intracran* OR intracerebral OR supratentorial OR infratentorial OR vertebrobasilar OR vertebrovascular) NEAR/5 (isch*emi* OR infarct* OR thrombo* OR emboli* OR occlus*)):ti,ab	186,630
#11	stroke*:ti,ab OR poststroke:ti,ab OR 'post stroke':ti,ab OR cerebrovasc*:ti,ab OR 'brain vasc*':ti,ab OR 'cerebral vasc*':ti,ab OR apoplex*:ti,ab OR sah:ti,ab	538,420
#10	'cerebro vascular*':ti,ab	1,886
#9	cerebrovascular*:ti,ab	90,333
#8	'cerebrovascular accident':ti,ab	7,918
#7	'stroke'/exp	369,803
#6	'cerebrovascular disorders'/exp	817,315
#5	(#1 OR #2 OR #3 OR #4) AND [1966-2021]/py AND [1-1-1966]/sd NOT [27-6-2021]/sd	2,702,927
#4	(('admission*' OR 're*admission*' OR 'stay*') NEAR/5 ('multiple' OR 'repeat*' OR 'hospital*')):ti,ab	319,741

#3	((**hospital** OR **patient**) NEAR/5 **readm***):ti,ab	27,575
#2	(**re*adm***:ti,ab OR **re*hospital***:ti,ab OR **re*attend***:ti,ab OR **re*admittan***:ti,ab OR **re*presentation**:ti,ab OR **admission***:ti,ab OR **hospitalization**:ti,ab OR **hospitalisation**:ti,ab OR **treatment**:ti,ab) AND **outcome**:ti,ab OR **revisit***:ti,ab OR **represent***:ti,ab OR **unplanned**:ti,ab OR **return**:ti,ab	2,569,355
#1	'**patient readmission**'/exp OR '**patient readmission**'	78,141
