# Supplementary material for: Rapid systematic review of readmissions costs after stroke
Source: Cost Eff Resour Alloc. 2024 Mar 12;22:22. doi: 10.1186/s12962-024-00518-3 (PMC10936094; doi:10.1186/s12962-024-00518-3)
Supplement: Supplementary file 2 — Supplementary Material 2 [file 12962_2024_518_MOESM2_ESM.pdf]

Appendix Supplemental Table 2 – Readmission cost and study approach classifications and definitions

**Readmissions cost**	
Readmission cost per index-hospitalization surviving patient	Total cost of readmissions / number of index-hospitalization patients
Readmission cost per patient	Total cost of readmissions / number of study patients
Readmission cost per readmitted patient	Total cost of readmissions / number of readmitted patients
Cost per readmission	Total cost of readmissions / number readmissions
**Study analytic economic perspectives***	
Healthcare payer and/or provider	Includes only monetary costs incurred by a (typically third party) healthcare payer (e.g., Medicare/ Medicaid, British national health service, a health maintenance organization).
Healthcare sector	Includes all monetary costs of healthcare, regardless of who bears the cost. This perspective also includes patients' out-of-pocket costs.
Limited societal	Accounts for cost components beyond those captured by the healthcare sector perspective, including patient time, patient transportation, unpaid caregiver time, and productivity loss.
Societal	Accounts all resources that could be used for other purposes, including the cost impacts affecting at least one of these other sectors: environment, education, or the justice system.
**Cost-of-illness studies epidemiological approach****	
Incidence-based	This approach estimates the present and future cost-of-illness in a given year.
Prevalence-based	This approach generally measures of cost-of-illness in the present and the past in a given year.
**Estimation procedure approach****	
Bottom-up	In this approach the estimation of costs can be divided into two steps: the first step is to estimate the quantity of health inputs used and the second step is to estimate the unit costs of the inputs used in medical our health care services. The costs are then estimated by multiplying unit costs by the quantities used.
Top-down	This estimation measures the proportion of a disease that is due to exposure to the disease or the risk factors. This kind of approach allocates portions of a known total expenditure to each of several broad disease category.
**Calculation Methods*****	
Partial economic evaluations	*These economic studies are a form of analysis that only examines the cost of an intervention or disease.*
Health expenditure study	A health expenditure focuses specifically on the costs related to healthcare services and products (such as hospitalization and medication expenses).

Cost-of-illness study	A cost-of-illness (COI) studies estimates and evaluates the direct and indirect costs of stroke. A COI study is limited as it only considers the cost and does not relate the costs of a certain intervention to the effects.
Full-economic evaluation	*These studies are a form of comparative economic analysis that evaluates two or more policy alternatives in terms of their relative costs and outcomes.*
Cost-effectiveness study	This study is a form of comparative economic analysis where the outcomes are measured in natural units (e.g. life-years gained, disease case averted).
Cost-utility study	This analysis evaluates two or more policy alternatives, and the outcomes are expressed by a generic measure of utilities that considers that considers both the effect on mortality and morbidity (e.g., quality-adjusted life-years (QALYs) and disability-adjusted life-years (DALYs)).
Cost-benefit study	This study is a comparative economic analysis where both the costs and outcomes are expressed in pecuniary terms (it should value the interventions relevant costs and outcomes based on the preferences of those affected (e.g., the individuals' willingness to pay)).
Cost-minimization study	This study is a form of analysis that compares the costs of two alternatives which are all assumed to have equivalent health effects.
Cost-consequence study	This analysis a comparative economic analysis in terms of relative costs and outcomes, where the outcomes are not summarized in a single measure, and multiple outcomes of interest are reported.
**Cost definitions********	
Direct costs	Costs incurred by the health system, society, family and individual patient, the direct costs consist of direct healthcare costs and non-healthcare costs.
Direct health-care costs	Costs related to medical care expenditures (e.g., costs of diagnosis, treatment/medications, or rehabilitation).
Non-health care costs	This defines costs related to the consumption of non-healthcare resources (e.g., transportation, household expenditures, informal cares of any kinds).
Indirect costs	This cost refers to productivity losses due to morbidity and mortality, borne by the individual, family, society, or the employer.
Non-health consequences (costs)	This term captures both medical and non-medical resources consumed, including patient time, transportation costs, caregiver time, productivity, and other non-healthcare sector impacts on education, criminal justice, housing, and environment.

* Adapted from reference 19

**Adapted from the following references: 17 and 18

*** Adapted from the following references: 18 and 22

**** Adapted from reference 17
