# Supplementary material for: Rapid systematic review of readmissions costs after stroke
Source: Cost Eff Resour Alloc. 2024 Mar 12;22:22. doi: 10.1186/s12962-024-00518-3 (PMC10936094; doi:10.1186/s12962-024-00518-3)
Supplement: Supplementary file 3 — Supplementary Material 3 [file 12962_2024_518_MOESM3_ESM.pdf]

Appendix Supplemental Table 3 – Study description of the time of follow-up, population characteristics and study approach.

Study, Country, [Ref.]	Cohort Period	Time Follow-up	Population characteristics		Study Approach			
			Stroke Type		Study design	Calculation Method (epidemiological approach if COI)	Economic perspective	Estimation procedure
Bjorkdahl, Sweeden, [27]	2004	3 weeks, 3 and 12 months	IS/ICH	First-ever	Randomized Controlled Trial, longitudinal	Cost-of-illness (prevalence-based)	Limited societal	Bottom-up
Brüggenjürgen, Germany, [38]	2000-2001	1 year	TIA/IS/ICH	First-ever/recurrent	Prospective	Cost-of-illness (prevalence-based)	Limited societal	Bottom-up
Cadilhac, Australia, [55]	1999-2004	5 years	IS/ICH/Und	First-ever	Prospective	Cost-of-illness (incidence-based)	Limited societal	Bottom-up
Caro, Canada, [37]	1990-1995	5 years	IS	First-ever/recurrent	Retrospective	Health expenditure	Health care payer	Top-down
Carod-Artal, Spain, [39]	1999-1997	1 year	IS/ICH	First-ever/recurrent	Prospective	Health expenditure	Health care payer	Bottom-up
Chang, Taiwan, [40]	1997-2002	1 year	TIA/IS/ICH/SAH	First-ever	Retrospective observational	Cost-effectiveness	Health care payer	Bottom-up
Chevreul, France, [31]	2007	1 year	TIA/IS/ICH/SAH	First-ever/recurrent	Prospective	Cost-of-illness (prevalence-based)	Limited societal	Both
Christensen, Scotland, [56]	2004-2005	1 year	IS/ICH	First ever	Retrospective cost study	Health expenditure	Health care payer	Top-down
Claesson, Sweden, [41]	1993-1994	1 year	IS/ICH	Not reported	Randomized cost-minimization trial	Cost-minimization	Limited societal	Bottom-up
Claesson, Sweden, [42]	1993-1994	1 year	IS/ICH	Not reported	Randomized prospective	Cost-of-illness (incidence-based)	Limited societal	Bottom-up
Deutschbein, Germany, [26]	2014-2015	1 year	TIA/IS/ICH/SAH	First-ever/recurrent	Non-randomized prospective	Health insurance expenditure	Health care payer	Bottom-up
Dewey, Australia, [57], [58]	1996-1997	1 year	IS/ICH/Und	First-ever	Prospective	Cost-of-illness (incidence-based)	Limited societal	Bottom-up
Fattore, Italy, [43]	2005-2007	1 year	IS/ICH	First-ever/recurrent	Observational, prospective, incidence-based multi-center	Cost-of-illness (incidence-based)	Limited societal	Bottom-up
Fjaertoft, Norway, [44]	1995-1997	1 year	IS/ICH/Other	Not reported	Randomized cost-minimization prospective trial	Health Expenditure	Health care provider	Top-down
Gerzeli, Italy [45]	NC (1998-1999)	3, 6 and 12 months	IS/ICH	First-ever	Observational, prospective, incidence-based multi-centre	Cost-of-illness (prevalence-based)	Limited societal	Bottom-up

Ghatnekar, Sweden, [60]	1997-2000	4 years	IS/ICH	First-ever	Prospective, incidence-based	Cost-of-illness (incidence-based)	Limited societal	Bottom-up
Ghatnekar, Sweden, [59]	2001	2,5-3 year	IS/ICH	First-ever	Prospective, incidence-based	Health expenditure	Health care provider	Top-Down
Ghatnekar, Sweden, [61]	2009-2011	2,5-3 year	IS/ICH/Und	First-ever	Prospective	Cost-of-illness (incidence-based)	Limited societal	Bottom-up
Gloede, Australia, [62]	1997-1999	3, 5 and 10 years	IS/ICH/Und	First-ever	Prospective	Cost-of-illness (incidence-based)	Limited societal	Bottom-up
Goeree, Canada, [46]	2001-2002	1 year	TIA/IS/ICH	First-ever	Prospective, multicentre	Cost-of-illness (prevalence-based)	Limited societal	Bottom-up
Hellsten, Canada, [29]	2008-2012	1 and 3 months	IS/ICH	First-ever	Retrospective observational	Health expenditure	Health care payer	Top-down
Hoffmann, USA, [68]	2010-2014	1 month	ICH	First-ever	Retrospective observational	Health expenditure	Health care payer	Top-down
Johnson, USA, [30]	2008-2013	1 year	IS	First-ever	Retrospective observational	Health medicare and insurance expenditure	Health care payer	Top-down
Lee, Taiwan, [47]	1997-2002	1 year	TIA/IS/ICH/SAH	First-ever	Retrospective observational	Health insurance expenditure	Health care payer	Bottom-up
Lee, USA, [35]	1997-2001	4 years	IS/ICH/SAH	First-ever	Retrospective observational	Health expenditure	Health care payer	Top-down
Luengo-Fernandez, England, [63]	2003-2007	1, 6, 12, 24 and 60 months	TIA/IS/ICH/SAH/ Und	First-ever	Retrospective and prospective	Health expenditure	Health care payer	Top-down
McGuire, Scotland, [36]	1995-2005	11 years	IS/ICH	First-ever	Retrospective population-based inception cohort	Health expenditure	Health care payer	Top-down
Meretoja, Finland, [48]	1999-2007	10 years	IS/ICH/SAH	First-ever	Prospective	Cost-of-illness (incidence-based)	Health care sector	Bottom-up
Mills, USA, [25]	1975	1 year	IS/ICH	First-ever	Population-based studies incidence-based cohort	Cost-of-illness (prevalence-based)	Limited societal	Top-down
Osberg, USA, [49]	1984	1 year	Stroke (Not clear)	First-ever	Prospective longitudinal	Health expenditure	Health care provider	Bottom-up
Persson, Sweden, [50]	1983-1985	10 months and 2 years	IS/ICH	First-ever	Prospective	Cost-of-illness (incidence-based)	Limited societal	Bottom-up
Porsdal, Denmark, [33], [64]	1994-1995	1 year	TIA/IS/Und	First-ever	Prospective	Cost-of-illness (prevalence-based)	Limited societal	Bottom-up

Rossnagel, Germany, [34]	2000-2001	1 year	TIA/IS/ICH/SAH	First-ever	Prospective	Cost-of-illness (prevalence-based)	Limited societal	Bottom-up
Spieler, France, [65], [66]	1994-1996	12 and 18 months	IS	First-ever/recurrent	Prospective	Health expenditure	Health care sector	Both
Stein, USA, [67]	2013	30 days	IS	First-ever/recurrent	Retrospective observational	Health expenditure	Health care payer	Top-down
Stowers, USA, [28]	2009-2014	7 and 30 days	TIA	First-ever	Retrospective observational	Health expenditure	Health care payer	Top-down
Taylor, USA, [51]	1992-1995	1 year	IS/HS	First-ever	Retrospective observational	Health medicare expenditure	Health care payer	Top-down
Tay-Teo, Australia, [52]	2004	1 year	IS/ICH	First-ever	Phase II, cost-effectiveness randomized controlled trial	Cost-effectiveness	Limited societal	Bottom-up
Teng, Canada, [53]	1997-1998	1 and 3 months	IS/ICH	First-ever	Randomized controlled cost-benefit prospective trial	Cost-benefit	Health care payer/provider	Both
Thorngren, Sweden, [54]	1986-1987	3, 6 and 12 months	IS/ICH/Und	First-ever/recurrent	Prospective	Health expenditure	Health care payer/provider	Bottom-up
Torbica, Italy [32]	2007-2008	3, 6 and 12 months	IS/ICH	First-ever/recurrent	Observational, prospective, incidence-based multi-centre	Cost-of-illness (prevalence-based)	Limited societal	Bottom-up

TIA, Transient Ischemic Accident; IS, Ischemic Stroke; ICH, Intracerebral Hemorrhage; HS, Hemorrhagic Stroke; SAH, Subarachnoid Hemorrhage; Und, Undetermined stroke; NC, Not Clear; NR, Not Reported; NRead, Not Readmitted; Read, Readmitted; USA- United Sates of America
