# Supplementary material for: Rapid systematic review of readmissions costs after stroke
Source: Cost Eff Resour Alloc. 2024 Mar 12;22:22. doi: 10.1186/s12962-024-00518-3 (PMC10936094; doi:10.1186/s12962-024-00518-3)
Supplement: Supplementary file 4 — Supplementary Material 4 [file 12962_2024_518_MOESM4_ESM.pdf]

Appendix Supplemental Table 4 – Overall stroke readmission costs

Study characterization			Readmission characterization		Costs description		
Study, Country, [Ref.]	Stroke Type	Sample size for economic analyses	n (%) of readmissions	Readmission Type	Readmission cost type<br>(Year price)<br>Reported cost (SD or IQR)	2021 US$ Cost PPP values**	Direct / Total (direct+indirect)<br>% of total costs
Bjorkdahl, Sweeden [27]	IS/ICH	58<br>(IS 38, ICH 20)	18 (31)	Planned and unplanned	(2004)		
				All-cause	Mean 12-month per readmission		
					2076 EUR	2956	-
Brüggenjürgen, Germany [38]	TIA/IS/ICH	367<br>(TIA 50, IS 139, ICH 11)	64 (17)	Planned and unplanned	(2005)		
				All-cause	Mean 12-month per index-hospitalization surviving patient		
					966 (3503)	1578 (5723)	10.3/ - *
Cadilhac, Australia, [55]	IS/ICH/Und	Estimated 31,951<br>(IS 27,660, ICH 4291)	Not reported	Planned and unplanned	(2004)		
				Stroke-complications	mean 12-month per index-hospitalization surviving patient		
				IS/Und	1442 AUSD	1542	5.8 / -
				ICH	482 AUSD	516	1.6 / -
					Weighted mean 12-month per index-hospitalization surviving patient*		
					1313 AUSD	1404	5.2 / -
				Stroke-recurrence	mean 12-month per index-hospitalization surviving patient		
				IS/Und	801 AUSD	857	3.2 / -
				ICH	460 AUSD	492	1.6 / -
					Weighted mean per index-hospitalization surviving patient *		
					774 AUSD	828	2.9 / -
Caro, Canada, [37]	IS	18,695	13,591 (72.7)	Unplanned	(2002)		Not clear

			(per 5-years)				
				Cardiovascular disease	Total annual cost		
					24,000,000 CAD	28,959,889	
					Mean 12-month per index-hospitalization surviving patient *		
					1284 CAD	1549	
				Bleeds	Total annual cost		
					5,000,000 CAD	6,033,310	
					Mean 12-month per index-hospitalization surviving patient *		
					267 CAD	322	
Carod-Artal, Spain, [39]	IS/ICH	90 (IS 89, ICH 11)	24 (26.6)	Planned and unplanned	(1997)		
				All-cause	Mean 12-month per index-hospitalization surviving patient		
					65,193 PST	969	7.3 / - *
				Stroke-recurrence	Mean 12-month per index-hospitalization surviving patient		
					6968 PST	104	0.78 / - *
Chang, Taiwan, [40]	TIA/IS/ICH/SAH/Und	2368 (TIA 686, IS 1180, ICH 424, SAH 78)	1207 (51) (death/readm.)	Planned and unplanned	(2002)		
				All-cause	Mean 12-month per patient		
				All-stroke	50,152 NTD	3544	29.0 / -
				< 65 years-old	37,655 NTD	2661	-
				65-74 years-old	44,978 NTD	3178	-
				75-79 years-old	67,707 NTD	4785	-
				>79 years-old	80,302 NTD	5675	-
			IS 573 (48.6 of IS), TIA 321 (48.6 of TIA)	IS (and TIA)			
			411 (38.4)	Mild to moderate stroke	33,022 NTD	2263	31.4 / -
			483 (60.8)	Severe stroke	72,537 NTD	5126	34.1 / -
					Weighted mean 12 month per patient *		

					49,857 NTD	3523	29.1 / - *
			ICH 267 (63 of ICH), SAH 46 (59 of SHA)	HS			
			175 (57.2)	Mild to moderate stroke	40,396 NTD	2855	26.0 / -
			138 (70.4)	Severe stroke	68,360 NTD	4831	18.1 / -
					Weighted mean per patient *		
					51,514 NTD	3640	22.9 / - *
Chevreul, France, [31]	TIA/IS/ICH/SAH	524,753 (120,982 incident cases stroke 93,571, TIA 27,411)	9549 (9)*	Planned and unplanned	(2007)		
				All-cause	Total first-year cost Incident cases		
					12,600,000 EUR	18,605,896	
					Mean 12-month per index-hospitalization surviving patient		
					1427 EUR	2107	7.4 / - *
Christensen, Scotland, [56]	IS/ICH	5311 (IS 4295, ICH 1016)	1867 (44.6)*	Planned and unplanned	(2006)		
				Stroke-recurrence/ cardiovascular disease	Mean 12-month per patient		
			IS 1624 (44.8)*	IS	3127 (8101) GBP	5926 (15,353)	22.3 / -
			ICH 243 (43.6)*	ICH	1918 (7248) GBP	3635 (13,737)	13.7 / - *
					Weighted mean per patient*		
					2896 GBP	5604	20.6 / - *
				Stroke-recurrence/ cardiovascular disease	Mean 12-month per patient readmitted		
				IS	8269 (11,449) GBP	15,672 (21,699)	-
				ICH	8011 (13,089) GBP	15,183 (26,171)	-
					Weighted mean per patient readmitted*		
					8220 GBP	15,579	-
				Stroke-recurrence/ cardiovascular disease	Mean 12-month per index-hospitalization surviving patient		

				IS	3702 (8693) GBP	7016 (16,475)	-
				ICH	3495 (9507) GBP	6624 (18,018)	-
					Weighted mean per index-hospitalization surviving patient *		
					3674	6962	26.2 / -*
				Stroke-recurrence/ cardiovascular disease	Mean 12-month per readmission		
				IS	4487 (7669) GBP	8504 (14,535)	-
				ICH	4022 (8442) GBP	7623 (15,600)	-
					Weighted 12-month mean per readmission *		
					4425 GBP	8386	-
Claesson, Sweden, [41]	IS/ICH	245 (166 stroke unity, 83 general ward) (NR)	Not reported	Planned and unplanned	(1996)		
				All-Cause	Mean 12-month per index-hospitalization surviving patient		
				Stroke-unity group	17,879 SEK	3084	10.5 / - *
				General ward group	19,198 SEK	3311	10.0 / - *
					Weighted mean 12-month per index-hospitalization surviving patient *		
					18,318 SEK	3160	10.3/ - *
				All-cause	Mean 12-month per index-hospitalization surviving patient		
				Mild Stroke	20,842 SEK	3595	19.4 / - *
				Moderate Stroke	23,283 SEK	4015	5.9 / - *
				Severe Stroke	11,656 SEK	2010	5.1 / - *
					Weighted mean per index-hospitalization surviving patient *		
					18,318 SEK	3160	10.3 / - *
				All-cause	Mean 12-month per surviving patient at the end of study		
				Mild Stroke	14,020 SEK	2418	13.9 / -*
				Moderate Stroke	23,088 SEK	3982	8.2 / - *

				Severe Stroke	20,022 SEK	3488	6.1 / - *
					Weighted mean per surviving patient *		
					17,595 SEK	3035	8.3 / -*
Claesson, Sweden [42]	IS/ICH	149 (No-Cognitive Impairment, 42 Cognitive Impairment 107) (IS 142, ICH 7)	Not reported	Planned and unplanned	(1996)		
				All-cause	Mean 12-month per index-hospitalization surviving patient		
				No-cognitive Impairment	7175 SEK	1237	9.7 / - *
				Cognitive Impairment	31,917 SEK	5505	13.5 / - *
					Weighted mean 12-month per index-hospitalization surviving patient *		
					24,943 SEK	3953	13.1 / - *
Deutschbein, Germany, [26]	TIA/IS/ICH/SAH	91 (31 IG, 60 SC)	46 (50.5)* (12-month)	Planned and unplanned	(2015)		Not reported
				All-cause	Median 6-month per readmission		
			12 (37.4)	Intervention group	0 (0 -1910) EUR	0 (0 - 2726)	
			34 (57.4)	Standard care	983 (0 - 4375) EUR	1339 (0 - 6224)	
Dewey, Australia, [57]	IS/ICH/Und (first-ever)	263 (IS 199, ICH 40, Und 24)	Not reported	Planned and unplanned	(1997)		
				Stroke-related	Mean 12-month per index-hospitalization surviving patient		
					973 AUD	1257	- / 5.1 *
				Stroke -recurrence	Mean 12-month per index-hospitalization surviving patient		
					576 AUD	744	- / 3.0 *
Dewey, Australia, [58]	IS/ICH/Und (first-ever)	127 (NR)	Not reported	Planned and unplanned	(1997)		
				Stroke-related	Total annual readmission cost		
					28,000,000 AUD	36,183,587	
				Stroke-recurrence	Total annual readmission cost		
					17,000,000 AUD	21,968,606	

				Stroke-related	Mean 12-month per index-hospitalization surviving patient *		
					956 AUD	1235	- / 5.0
				Stroke-recurrence	Mean 12-month per index-hospitalization surviving patient *		
					580 AUD	750	- / 3.0
Fattore, Italy, [43]	IS/ICH	411 (IS 352, ICH 59)	43 (10.5)	Planned and unplanned	(2007)		
				All-cause	Mean 12-month per index-hospitalization surviving patient		
					439 (1693) EUR	755 (279)	3.7 / 2.2 *
Fjaertoft, Norway, [44]	IS/ICH/other	320 (160 each group) (Not reported)	Not reported	Planned and unplanned	(1997)		
				All-cause	Mean 12-month per index-hospitalization surviving patient		
				Intervention group	2532 (0-524,428) EUR	5301(0-109,810)	13.4 / - *
				Standard care	3188 (0-27,098) EUR	6775(0-56,735)	14.6 / - *
					Weighted mean 12-month per index-hospitalization surviving patient *		
					2860 EUR	4909	14.0 / - *
Gerzeli, Italy [45]	IS/ICH	386 (Not reported)	21 (5.4)	Planned and unplanned	(1998)		
				All-cause	Mean 6-month per index-hospitalization surviving patient		
					259 EUR	531	- / 2.2
Ghatnekar, Sweden, [60]	IS/ICH (first-ever)	4357 (2187 men, 2170 women) (Not reported)	Not reported	Unplanned	(2000)		
				Stroke-recurrence	Mean 12-month per patients surviving 4-years		
				Year 1			
				Male	8709 (35,006) SEK	1431 (5753)	6.1 / - *
				Female	8310 (32,776) SEK	1364 (5386)	5.8 / - *

					Weighted mean per year 1 survivors*		
					8514 SEK	1399	6.0 / - *
				Year 2			
				Male	4449 (22,162) SEK	731 (3642)	9.2 / - *
				Female	4467 (19,528) SEK	734 (3209)	7.4 / - *
					Weighted mean per year 2 survivors *		
					4458 SEK	723	8.9 / - *
				Year 3			
				Male	3279 (16,565) SEK	538 (2722)	6.2 / - *
				Female	3921 (20,838) SEK	644 (3424)	6.4 / - *
					Weighted mean per year 3 survivors*		
					3591 SEK	590	6.3 / - *
				Year 4			
				Male	2131 (14,634) SEK	350 (2402)	4.4 / - *
				Female	1665 (1034) SEK	273 ()	3.1 / - *
					Weighted mean per year 4 survivors *		
					1895 SEK	170	3.7 / - *
Ghatnekar, Sweden, [59]	IS/ICH<br>(first-ever)	6611<br>(Not reported)	529 (8)<br>(12-month)	Unplanned	(2001)		Not reported
				Stroke-recurrence	Mean 12-month per patient		
					8147 (4512) EUR	12,081 (6690)	-
Ghatnekar, Sweden, [61]	IS/ICH/Und<br>(first-ever)	13,545<br>(4481(year 1997), 9064 (year 2009))<br>(1997: IS 3652, ICH 515, Und 313;<br>2009: IS 7659 ICH 1179 Und 226)	Not reported	Planned and unplanned	(2009)		
				1997 Cohort			
				Stroke-readmission	Mean 12-month per patient		
				Year1			
				Male	1112 EUR	1630	7.0 / 5.8 *
				Female	1061 EUR	1555	6.5 / 6.0 *
					Weighted mean per patient*		

					1087 EUR	1593	6.7 /5.9 *
				Year 2			
				Male	470 EUR	689	13.8 / 7.5 *
				Female	447 EUR	655	10.2 / 7.9 *
					Weighted mean per surviving patient*		
					459 EUR	673	11.8 /7.7 *
				Year 3			
				Male	317 EUR	465	9.1 / 5.3 *
				Female	365 EUR	535	8.7 / 6.9 *
					Weighted mean surviving patient *		
					341 EUR	500	8.9 / 6.2 *
				2009 Cohort			
				All-cause	Mean 12-month per patient		
				Year1			
				Male	300 EUR	440	2.0 / 1.6 *
				Female	245 EUR	359	1.6/ 1.4 *
					Weighted mean per patient *		
					273 EUR	400	1.8 / 1.5 *
				Year 2			
				Male	188 EUR	276	4.2 / 2.6 *
				Female	208 EUR	305	3.3 / 2.7 *
					Weighted mean per surviving patient *		
					198 EUR	290	3.7 / 2.7 *
				Year 3			
				Male	112 EUR	155	3.1 / 1.8 *
				Female	73 EUR	107	1.5 / 1.2 *
					Weighted mean per surviving patient*		
					94 EUR	138	2.2 / 1.5 *
Gloede, Australia, [62]	IS/ICH/Und	286 (IS 243, ICH 43)	Not reported	Planned and unplanned	(2010)		

				Stroke-recurrence	Mean 12-month per readmission (first-year)		
				IS	32,354 AUD	26,788	Not reported
				ICH	32,354 AUD	26,788	Not reported
				Stroke-complications	(3–5 years) 12-month mean per index-hospitalization surviving patient		
				IS	117 USD	150	2.2 / -
				ICH	183 USD	223	3.1 / -
					(3–5 years) weighted 12-month mean per index-hospitalization surviving patient *		
					127 USD	158	2.3 / - *
				Stroke-complications	(10 years)12-month mean per index-hospitalization surviving patient		
				IS	108 USD	132	2.1 / -
				ICH	26 USD	32	0.3 / -
					(10 years) weighted 12-month mean per index-hospitalization surviving patient *		
					96 USD	120	1.72 / -
Goeree, Canada, [46]	TIA/IS/ICH	365 (TIA 135, IS 188, ICH 42)	Not reported	Planned and unplanned	(2004)		
				All-cause	Mean 12-month per patient		
				TIA	8095 CAD	9161	- / 46.0
				IS	11,162 CAD	12,632	- / 21.0
				ICH	5218 CAD	5905	- / 9.0
					Weighted mean per patient*		
					9344 CAD	10,574	- / 23.0 *
Hellsten, Canada, [29]	IS/ICH	37,028 (IS 29724 (30 days 28642, 90 days 27863), ICH 7304 (30 day 7014, 90 days 6845)	6522 (17.6) (90 day overall)	Planned and unplanned	(2012)		
			3189 (8.6) (30-day overall)	All-cause	Mean per (4 years) 30-day per index-hospitalization surviving patient		
			2635 (9.2)*,	IS	677 (451-872) CAD	642 (423-823)	2.8 / -
			554 (7,9)*	ICH	715 (365-1410) CAD	679 (679-1338)	2.8 / -

					Weighted mean per patient per patient surviving index-hospitalization*		
					685 CAD	650	2.8 / - *
					Mean per (4 years) 30-day per readmission		
				IS	8145 (5136-9107) CAD	7729 (4874-8642)	
				ICH	10,035 (6208-13,884) CAD	9523 (5891-13,175)	
					Weighted mean per (4 years) 30-day per readmission*		
					8518 CAD	8083	
			6522 (17.6)<br>(90 day overall)	All-cause	Mean per (4 years) 90-day mean per patient surviving index-hospitalization		
			5461 (19.6)*,	IS	2060(1430-2924) CAD	1955 (1357-2775)	6.6 / -
			1061 (15,5)*	ICH	1898 (1430-2924) CAD	1801 (1357-2775)	6.1 / -
					Weighted mean per (4 years) 90-day per patient surving index-hospilization*		
					2028 CAD	1925	6.5 / - *
					Mean per (4 years) 90-day per readmission		
				IS	13,068 (10,308-16,142) CAD	12401 (9782-15,318)	
				ICH	15,524 (9656-20,839) CAD	14732 (9163-19,775)	
					Weighted mean per (4 years) 90-day per readmission *		
					13,553 CAD	12,861	
Hoffmann, USA, [68]	ICH	64,609	8372 (12.9)<br>(30 day rate/5 years)	Planned and unplanned	(2019)		Not reported
				All-cause	Five-year total readmission cost		
					160,320,059 USD	167,069,927	
			8372 (12.9)	All-cause	Median 30-day cost of individual readmissions (5 year)		
					10,342 (5641-19,584) USD	10,777 (5879-20,409)	

Johnson, USA, [30]	IS	51,251<br>(Medicare 31037)	12,042 (39.0)<br>Medicare sample	Planned and unplanned	(2013)		
				Stroke-complications	30-day mean readmission cost		
					12,000 USD	13,807	Not clear
				Stroke-complications<br>(medicare)	30-day total *		
					144,504,000 USD	1,666,260,192	15.1 / - *
Lee, USA, [35]	IS/ICH/SAH	11,430<br>(IS 9131, ICH 1757, SAH 342)	6936 (60.7)*<br>(per four years)<br>Medicare sample	Planned and unplanned	(2001)		
				All-cause	Mean per year (four-years) per patient*		
			5743* (62.9)	IS	11,895 USD	17,453	30.1 / - *
			1009* (60.9)	ICH	11,601 USD	17,022	30.5 / - *
			184* (53.6)	SAH	14, 545 USD	21,342	30.1 / - *
					Weighted mean per year (4 years) per patient*		
					11,721 USD	17,198	30.2 / - *
Lee, Taiwan, [47]	TIA/IS/ICH/SAH/Und	2368<br>(TIA 686, IS 1180, ICH 424, SAH 78)	923 (43.3)	Planned and unplanned	(2002)		
				All-cause	Mean 12-month per patient		
				All-stroke	50,164 NTD	3545	29.0 / - *
			290 (43.7)	TIA	43,157 NTD	3037	6.2 / - *
			482 (43.5)	IS	53,541NTD	3768	23.7 / - *
			142 (44.7)	ICH	58,278 NTD	4102	35.6 / - *
			9 (22.0)	SAH	13,423 NTD	945	31.9 / - *
Luengo-Fernandez, England, [63]	TIA/IS/ICH/SAH/Und	734<br>(Stroke 439, TIA 295)	NR (8)	Planned and unplanned	(2009)		
				Vascular complications	Mean 12-month per index-hospitalization surviving patient		

				(within 7 days of subsequent vascular events)			
			0.18 per patient/ year	IS/ICH/SAH/Und	653 (4126) GBP	1155 (7297)	9.9 / - *
			0.17 per patient/year	TIA	720 (3252) GBP	1273 (5751)	29.8 / - *
					Weighted mean per index-hospitalization surviving patient *		
					680 GBP	1203	13.8 / - *
McGuire, Scotland, [36]	IS/ICH	9568<br>(IS 8893, ICH 705)	5401 (56.4)<br>per 11-years	Planned and unplanned	(2005)		
				All-cause	Mean per year (eleven-years) per patient		
				IS	10,262 (19,046) GBP	20,019 (37,155)	50.8 / - *
				ICH	7252 (18,396) GBP	14,147 (35,887)	44.5 / - *
					Weighted mean per year (eleven-years), per patient *		
					10,041 GBP	19,588	50.2 / - *
					Mean readmission cost per index-hospitalization surviving patient		
				IS	14,681 (21,307) GBP	28,640 (41,566)	54 / -
				ICH	13,349 (23,382) GBP	26,041 (45,614)	45 / -
					Weighted mean per year (eleven-years), per index-hospitalization surviving patient *		
					14,567 GBP	28,417	53.5 / - *
					Mean readmission cost per readmission		
				IS	3783 (8057) GBP	7380 (15,718)	
				ICH	3240 (7349) GBP	6321 (14,336)	
					Weighted mean per readmission (eleven-years)*		
					3750 GBP	7316	-
Meretoja, Finland, [48]	IS/ICH/SAH	94,316<br>(2007: 10,338 (IS 8204, ICH 1413, SAH 721)	Not clear	Planned and unplanned	(2008)		
				All-cause	Mean 12-month per patient – year 2007		
				IS	6913 USD	6486	23.4 / - *

				ICH	4703 USD	3821	13.0 / - *
				SAH	5339 USD	5009	12.5 / - *
					Weighted mean 12-month per patient – year 2007*		
					6501 USD	6099	19.4 / - *
Mills, USA, [25]	IS/ICH	13214 (7522 first-stroke survivors)	Not reported	Unplanned	(1975)		
				Stroke recurrence	Total readmission cost per first stroke survivor[+]		
					6,790,000 USD	31,148,987	3.3 / 1.8 *
				Stroke recurrence	Mean 12-month per first stroke survivor*		
					903 USD	4143	
Osberg, USA, [49]	Stroke (not clear)	89	29 (32.6)	Planned and unplanned	(1984)		
				All-cause	Mean 12-month charges per index-hospitalization surviving patient		
					6117 (11,724) USD	13,538 (25,948)	- / 13.0
					Mean 12-month charges per readmission		
					8120 USD	17,971	-
Persson, Sweden, [50]	IS/ICH	125 (IS 118, ICH 7)	Not reported	Planned and unplanned	(1985)		
				All-cause	Mean 12-month per patient (first-year)		
		60 Male		Male	4769 SEK	1394	5.5 / - *
		65 Female		Female	2590 SEK	757	2.8 / - *
					Weighted mean per patient*		
					3636 SEK	1063	3.8 / - *
				All-cause	Mean 12-month per surviving patient (second-year)		
		46 Male		Male	1164 SEK	340	2.8 / - *
		36 Female		Female	770 SEK	225	0.7 / - *

					Weighted mean per surviving patient*		
					991 SEK	290	-
Porsdal, Denmark, [33]	TIA	73	5 (7.8)	Planned and unplanned	(1995)		
				Stroke-related	Mean 12-month per index-hospitalization surviving patient		
					1650 DKK	360	8.8 / - *
Porsdal, Denmark, [64]	IS/Und	340	56 (16)	Planned and unplanned	(1995)		
				Stroke-related	Mean 12-month per index-hospitalization surviving patient*		
					9380 DKK	2043	- / 20.0
Rossnagel, Germany, [34]	TIA/IS/ICH	383 (TIA 30, IS 65, ICH 5)	68 (18)	Planned and unplanned	(2004)		
				Stroke-related	Mean 12-month per patient alive at the end of study (questionary responders)		
					993 (3544) EUR	1630 (5816)	- / 8.6 *
Spieler, France, [65]	IS	435	Not reported	Planned and unplanned	(1997)		
				All-cause	Total expenditure		
					2,541,44 EUR	446,459	
				All-cause	Mean 12-mont per index-hospitalization surviving patient		
					584 (445-724) EUR	1010 (770-1252)	3.3 / - *
Spieler, France, [66]	IS	435	Not reported	Planned and unplanned	(1997)		
		(first-ever 346, recurrent 89)		All-cause	Total 18-month expenditure		
				First-ever	227,779 EUR	397,692	
				Recurrent	63,119 EUR	110,203	
					Mean 18-month per index-hospitalization surviving patient		
				First-ever	658 (479-837) EUR	1149 (836-1461)	3.3 / - *
				Recurrent	709 (413-1005) EUR	1238 (721-1755)	3.8 / - *
					Weighted mean per index-hospitalization surviving patient*		
					668 EUR	1166	3.4 / - *

Stein, USA, [67]	IS	192,594	24,545 (12.7)*	Planned and unplanned	(2013)		
				All-cause	Mean 30-day per patient readmitted (unadjusted)		Not reported
			17,271 (8.9)*	Same hospital	51,087 (49,878-52,296) USD	52,054 (50,822-53,285)	
			7274 (3.7)*	Different hospital	61,633 (not clear) USD	62,798	
					Weighted mean 30-day per patient readmitted*		
					54,212 USD	55,238	
Stowers, USA, [28]	TIA	985,851		Planned and unplanned	(2014)		
			34,503 (3.7)*	All-cause	Mean 7-day per index-hospitalization surviving patient		Not reported
				Year 2009	9322 USD	10,526	
				Year 2010	Not clear		
				Year 2011	Not clear		
				Year 2012	Not clear		
				Year 2013	Not clear		
				Year 2014	10,961 USD	12,377	
			91,261 (9.8)		Mean 30-day per index-hospitalization surviving patient		
				Year 2009	9323 USD	10,528	
				Year 2010	9754 USD	11,014	
				Year 2011	10,735 USD	12,122	
				Year 2012	10,336 USD	11,671	
				Year 2013	10,822 USD	12,220	
				Year 2014	11,320 USD	12,783	
					Weighted mean per index-hospitalization surviving patient*		
					10,327 USD	11,661	
Taylor, USA, [51]	IS/HS	793	Not reported	Planned and unplanned	(1994)		
				All-cause	Mean 6-month (ten-years) per index-hospitalization surviving patient		
				For profit hospitals	2120 USD	3526	17.9 / - *

				Government hospitals	1719 USD	2859	18.9/ - *
				Non-profit hospitals	2531 USD	4210	19.9/ - *
				Minor teaching hospitals	2538 USD	3922	17.8/ - *
				Major teaching hospitals	2849 USD	4733	20.5/ - *
					Weighted 6-month (ten-years) mean per index-hospitalization surviving patient *		
					2415 USD	4017	19.4 / - *
Tay-Teo, Australia, [52]	IS/ICH	71 (SC 33, IG 38)		Planned and unplanned	(2004)		
			14 (19.7) *	All-cause	Mean 3-month per index-hospitalization surviving patient readmitted		
			8 (24)	Standard-Care	5337 (5432) AUD	5337 (5809)	- / 12.3 *
			6 (16)	Intervention-Group	4952 (4836) AUD	4975 (5172)	- / 36.5 *
					Weighted 3-month mean per index-hospitalization surviving patient readmitted*		
					5712 AUD	6109	- / 20.8 *
			28 (39.4)*	All-cause	Mean 12-month per index-hospitalization surviving patient readmitted		
			16 (48)	Standard-Care	7080 (8996) AUD	7571 (9621)	- / 14.5 *
			12 (32)	Intervention-Group	5493 (5342) AUD	5875 (5713)	- / 31.3 *
					Weighted 12-month mean per index-hospitalization surviving patient readmitted*		
					6231 AUD	6664	- / 19.5 *
Teng, Canada, [53]	IS/ICH	114 (SC 56, IG58)	13 (11.4)*	Unplanned	(1998)		
				All-cause	Mean 3-month per index-hospitalization surviving patient		
			3 (5,2)	Intervention-Group	364 (1795) CAD	481(2367)	4.7 / - *
			10 (17.9)	Standard-Care	1739 (5505) CAD	2368 (7268)	15.7 / - *
					Weighted mean 3-month per index-hospitalization surviving patient *		
					1039 CAD	1372	11.0 / - *
Thorngren, Sweden, [54]	IS/ICH/Und	258	43 (16.7)	Planned and unplanned	(1986)		

		(IS/Und 245, ICH 13)					
				All-cause	Weighted 12-month per index-hospitalization surviving patient *		
					7330 SEK	2127	6.9 /- *
Torbica, Italy [32]	IS/ICH	476 (IS 407, ICH 69)	51 (10.7)*	Planned and unplanned	(2008)		
				Stroke-Related	Mean per index-hospitalization surviving patient		
				With caregiver			
			10 (0.04)	0-3 month	198 (1222) EUR	319 (1969)	3.3 / - *
					Mean per surviving patient		
			13 (0.06)	3-6 month	207 (922) EUR	334 (1486)	12.7 / - *
			9 (0.04)	6-12 month	182 (948) EUR	293 (1528)	15.5 / - *
				Without caregiver			
				Stroke-Related	Mean per index-hospitalization surviving patient		
			5(0.02)	0-3 month	43 (317) EUR	69 (511)	3.3/ - *
					Mean per surviving patient		
			3 (0.01)	3-6 month	33 (329) EUR	53 (530)	12.7 / - *
			11 (0.05)	6-12 month	146 (768) EUR	235 (1238)	15.5 / - *

IRF, Inpatient Rehabilitation Facility; SNF, Skilled Nursing Facility; IG, Intervention Group; SC, Standard Care; Death/readm., death/readmission; TIA, Transient Ischemic Accident; IS, Ischemic Stroke; HS, Hemorrhagic stroke,; ICH, Intracerebral Hemorrhage; SAH, Subarachnoid Hemorrhage; Und, Undetermined stroke; NC, Not Clear; NR, Not Reported; USA, United Sates of America; AUSD, Australian Dollar; CAD, Canadian Dollar; DKK, Danish Krona; EUR, Euro; GBP, Great Britain Pound; NTD, New Taiwanese Dollars; PST, Pesetas; USD, US Dollar; SEK, Swedish Krona; PPP, Purchase Parity Prices

* Authors' calculation based on articles data

**Purchase Parity Prices calculated with CCEMG – EPPI-Centre Cost Converter (https://eppi.ioe.ac.uk/costconversion/default.aspx)

+After adjusting to 1980 prices (https://www.bls.gov/data/inflation_calculator.htm)
