# Supplementary material for: Rapid systematic review of readmissions costs after stroke
Source: Cost Eff Resour Alloc. 2024 Mar 12;22:22. doi: 10.1186/s12962-024-00518-3 (PMC10936094; doi:10.1186/s12962-024-00518-3)
Supplement: Supplementary file 5 — Supplementary Material 5 [file 12962_2024_518_MOESM5_ESM.pdf]

Appendix Supplemental Table 5 - Summary of the Reporting Quality (CHEERS checklist).

Number	Study, country, reference	1. Title	2. Abstract	3. Background and objectives.	4. health economic analysis	5. study population	6. Setting and location	7.comparators	8.perspective	9. Time horizon	10. Discount rate	11. Choice of health outcomes	12. measurment of outcomes	13. Valuation o outcomes	14. measurement and valuation of resources costs	15. Currency, price date, and conversion	16. Rational and description of model	17. Analytics and assumptions	18. Characterising heterogenity	19. Characterising distributional effects	20. Characterising uncertainty	21. Aproach to engagement with patients and others affected by the study	22. Study parameters	23. summary of main results	24. Effect of uncertainty	25. Effect of engagemnet with patients and others affect by study	26. Study findings, limitations, generalizability, and current knowledge	27. Source of funding	28. Stated conflicts of interest
1	Bjorkdahl, Sweden, 27	1	1	1 to 2	2 3	2 3	2 3	2 3	2	2	_	2 to 3	2 to 3	2 to 3	2 to 3	3	NA	2 to 3	4 6	6	6	NA	4 to 5	4 to 5	_	NA	5 to 8	8	8
2	Brüggenjürgen, Germany, 38	1	1	1 to 2	1 to 3	2 to 3	2 to 3	2	1	1	_	1 to 3	2 to 3	3 to 3	2 to 3	3	NA	1 to 3	5	_	3 to 4	NA	3 to 5	4 to 5	_	NA	5 to 6	6	_
3	Cadilhac, Australia, 55	1	1	1 to 2	1 to 2	2	2	2	1	5	2	2	2	2	2	2	NA	2	5 to 6	_	6	NA	3 to 5	5	7 8	NA	5 to 7	7	7
4	Caro, Canada, 37	1	1	2	2 to 3	2	2	_	_	2	_	2 to 3	2 to 3	2 to 3	3	3	NA	3	3	_	4	NA	3 to 4	3 to 4	_	NA	5 to 6	7	7
5	Carod-Artal, Spain, 39	1	1	1	1 to 2	1	1	_	1	1	_	1 to 2	2	2	2	_	NA	2	2	_	_	NA	3 to 4	3 to 4	_	NA	5 to7	_	_
6	Chang, Taiwan, 40	1	1	1 to 2	2	2	2	2	2	2	_	3	3	3	3 to 4	3	3 to 4	3 to 4	4 to 8	4 to 8	4 to 8	NA	4 to 8	4 to 8	6 to 7	NA	9 to10	10	10
7	Chevreul, France, 31	1	1	1 to 2	1 to 3	2	1	_	2	3	_	1 to 3	1 to 3	1 to 3	2 to 3	2	NA	2	2	4	_	NA	3 to 5	3 to 5	_	NA	5 to 6	6	6
8	Christensen, Scotland, 56	1	1	1 to 2	2	2	2	2 to 3	6	_	_	2	2	2	2	2	NA	2	3 to 5	3 to 5	_	NA	5 to 6	3	5 to 6	NA	5 to 6	7	_
9	Claesson, Sweden, 41	1	1	2	2 to 3	2	2	2	_	_	_	2	2	2	2 to 3	2	NA	3	6 to 8	_	_	NA	3 to 8	3 to 8	3 to 8	NA	4 to 7	7	_
10	Claesson, Sweden 42	1	1	2	2 to 3	2	2 3	2 3	_		_	2 to 3	2 to 3	2 to 3	2 to 3	3	NA	3	3 to 5	4 5	_	NA	3 to 6	3 to 6	_	NA	6 to 7	8	_
11	Deutschbein, Germany, 26	_	1	2	2 to 5	3	2 3	3	_	3	_	3	3	3	4 to 5	_	NA	4 to 5	6	6 to 8	6 to 9	NA	5 to 7	6 to 8	6 to 9	NA	7 to 10	10	10
12	Dewey, Australia, 57	1	1	1 to 2	2 to 3	2 to 3	2 to 3	3	2	_	2	2 to 3	2 to 3	2 to 3	2 to 3	3	NA	2 to 4	6	_	6	NA	3 to 6	4 to 5	5 to 7	NA	5 to 7	7	_
13	Dewey, Australia, 58	1	1	1	2	1 to 2	1 to 2	2	1	_	2	2 to 3	2 to 3	2 to 3	2 to 3	3	NA	2	5	3	5	NA	2 to 5	2 to 5	5 to 6	NA	5	5	_
14	Fattore, Italy, 43	1	1	2	2 to 3	2 to 3	2 to 3	_	2	_	_	3	3 to 3	3 to 3	3	6	NA	3 to 4	8	7 8	8	NA	4 to 5	4 to 6	9 to 10	NA	9 to 10	10	10
15	Fjaertoft, Norway, 44	_	1	1 to 2	2 to 3	2 to 3	2	2 to 3	6	_	_	2	2 to 3	2 to 3	2 to 3	5	NA	2 to 3	5	5	5	NA	4 to 5	4 to 5	7	NA	5 to 8	8	_
16	Gerzeli, Italy, 45	1	1	1 to 2	2 to 3	2 to 3	2 to 3	_	1	_	_	2 to 3	2 to 3	2 to 3	2 to 3	5	NA	3	6	5 6	6	NA	4 to 5	4	6	NA	6 to 7	7 to 8	_
17	Ghatnekar, Sweden, 60	1	1	1	1 to 2	1 to 2	1 to 2	_	1	_	2	1 to 2	2 to 3	2 to 3	2 to 3	2	NA	2	5	5	_	NA	2 to 3	2 to 3	4 to 6	NA	4 to 7	6	6
18	Ghatnekar, Sweden, 59	1	1	1 to 2	2 to 3	2 to 3	2 to 3	5	2	2	2	2 to 3	2 to 3	2 to 3	3	3 4	NA	2	4	4	_	NA	3 to 5	3 to 5	5	NA	5 to 6	_	_
19	Ghatnekar, Sweden, 61	1	1	1	2 to 3	1 to 2	2	2	2	2	2	2	2	2	2	2	NA	2	2 to 3	4	_	NA	2 to 4	3 4	5 to 6	NA	5 to 6	5	_
20	Gloede, Australia, 62	1	1	1	2	2	2	_	2	4	2	2	2	2	2	2	2	2	5	4	3	NA	2 to 3	3 to 5	3 to 5	NA	3 to 5	6	6
21	Goeree, Canada, 46	1	1	2	4 to 5	3	3	_	_	5	_	5 to 8	5 to 8	5 to 8	6 to 7	4	NA	7 to 8	9	11 13	_	NA	8 to 14	10	14 to 15	NA	14 to 17	14	_
22	Hellsten, Canada, 29	_	1	1 to 3	3 to 4	3	3	4 5	3	3	_	3 to 5	3 to 5	3 to 5	6 to 8	_	4 to 5	3 to 5	6 to 8	6 to 7	6 to 8	NA	6 to 8	6 to 10	_	NA	8 to 9	9	_
23	Hoffmann, USA, 68	_	1	1	1 to 2	2	1	_	_	2	_	2	2	2	2	2	NA	2	4	5 to 6	_	NA	6 to 8	6 to 8	3 to 7	NA	5 to 7	7	7
24	Johnson, USA, 30	1	1	1 to 2	3	2	2	3	2	2	_	2 to 3	2 to 3	2 to 3	2 to 3	3	NA	3	5 to 6	5 to 7	_	NA	3	5 to 6	6 to 7	NA	5 to 7	7	7
25	Lee, USA, 35	1	1	1 to 2	2	2	2	_	2	2	_	2	2	2	2	2	NA	2	3 4 5	4 to 6	_	NA	2	3 to 7	7 to 8	NA	7 to 8	8	_
26	Lee, Taiwan, 47	1	1	2	2	2	2	_	2	2	_	3 to 4	3 to 4	3 to 4	3 to 4	10	NA	4	6	6 to 8	_	NA	3, 6 to 9	3 to 9	9 to 10	NA	9 to 10	10	_
27	Luengo-Fernandez, England, 63	1	1	1	2	1	1	_	_	1	_	2	2	2	2	_	NA	2 3	3 to 5	5 to 7	_	NA	3 to 5	3 to 5	8	NA	5 to 8	_	_
28	McGuire, Scotland, 36	_	1	1 to 2	2	2	2	_	_	2	2	2	2	2	2	2	NA	2	2 to 3	4	_	NA	3	_	5	NA	4 to 7	7	_
29	Meretoja, Finland, 48	1	1	1	2	1 2	1	_	2	2	2	2	2	2	2	2	NA	2 3	3 to 5	4 to 5	_	NA	3 to 4	4	5	NA	4 to 6	6	6
30	Mills, USA, 25	1	_	2	2	3	2	2	2	2	2	2	2	2	2	2	NA	_	2	3 to 4	_	NA	2 to 4	_	3 4	NA	3 to 4	_	_
31	Osberg, USA, 49	1	1	1	2	2	2	_	_	_	_	2	2	2	2	2	NA	_	4 to 6	5	_	NA	4 to 6	4 to 5	6	NA	5 to 7	_	_
32	Persson, Sweden, 50	1	1	1 to 2	2 to 5	4	4	_	2	3	3	2 to 5	2 to 5	2 to 5	4 to 5	_	2 to 3	2 to 3	4	5 7 8 9 10	_	NA	5 to 10	5 7 8 9 10	_	NA	10 to 13	_	_
33	Porsdal, Denmark, 33	1	1	1	2 to 3	1	1	_	1	1	_	1 to 2	1 to 2	1 to 2	1 to 2	2	NA	2	3	_	_	NA	3 to 4	3	_	NA	3 to 4	_	_
34	Porsdal, Denmark, 64	1	1	2	1 to 4	2	2	_	3	2	_	2 to 4	2 to 4	2 to 4	3	3	NA	4	4 to 7	8 9	_	NA	4 to 9	9	_	NA	10 to 11	_	_
35	Rossnagel, Germany, 34	1	1	1	2	1	1	_	1	1	_	2	2	2	2	2	NA	2 to 3	3	_	_	NA	3 4	3 4	6	NA	5 to 6	6	_
36	Spieler, France, 65	1	1	2	2 to 3	2	2 to 3	_	3	_	_	3	2 to 3	2 to 3	2 to 3	3	NA	3	7	5 6	_	NA	3 to 4	5 to 8	_	NA	5 8 9	9	_
37	Spieler, France, 66	1	1	1	2 to 3	1 to 2	1 to 2	2	2	_	_	1 to 2	1 to 2	1 to 2	1 to 2	2	NA	2	3	3	_	NA	2 to 3	2 to 3	_	NA	2 to 3	3 to 4	_
38	Stein, USA, 67	_	1	2	2 to 3	2	2	_	_	2	_	2 to 3	2 to 3	2 to 3	3	_	3	3	4	6	6	NA	5 to 7	5 6 7	_	NA	3 to 7	7	_
39	Stowers, USA, 28	_	1	2	2	2	2	2	_	2	_	2	2	2	2	_	NA	2	2	3 to 4	_	NA	2 to 5	2 to 5	_	NA	5 to 6	_	_
40	Taylor, USA, 51	1	1	2	2 to 3	1 to 2	2	2	2	2	_	2	2 to 3	2 to 3	2 to 3	4	NA	3	3 to 4	3 to 4	3 to 5	NA	3 to 5	3 to 5	_	NA	5 to 6	6	_
41	Tay-Teo, Australia, 52	1	1	1 to 3	3	3	3	3	3	_	4	3	3	3	3	3	NA	3	5	4	5 to 6	NA	4 to 6	4 5	_	NA	4 to 6	_	_
42	Teng, Canada, 53	1	1	1 to 2	2	2	2	_	2	_	_	2	2	2	2	2	NA	2	3 4 5 6	3 4 5 6	5	NA	3 to 6	3 to 6	8	NA	7 to 9	_	_
43	Thorngen, Sweden, 54	_	1	1 to 2	2	2	2	_	2	2	_	2	2	2	2	2	NA	_	2 to 6	6	_	NA	2 to 6	3 to 6	_	NA	7 to 9	9	_
44	Torbica, Italy, 32	_	1	1 to 3	3 to 5	3	3	4 5	3	3	_	3 to 5	3 to 5	3 to 5	3 to 5	_	4 5	3 to 5	4 5	4 5	6 7	NA	4 5 6	4 5 6	7 to 8	NA	7 to 9	9	_
	N=	35	43	44	44	44	44	21	33	30	12	44	44	44	44	36	6	41	44	36	18	44	44	42	27	44	44	33	12
