# Supplementary material for: Rapid systematic review of readmissions costs after stroke
Source: Cost Eff Resour Alloc. 2024 Mar 12;22:22. doi: 10.1186/s12962-024-00518-3 (PMC10936094; doi:10.1186/s12962-024-00518-3)
Supplement: Supplementary file 6 — Supplementary Material 6 [file 12962_2024_518_MOESM6_ESM.pdf]

Appendix Supplemental Table 6 – Transient Ischemic attack readmission costs

Study characterization		Readmission characterization		Costs description		
Study, Country, [Ref.]	Sample size for economic analyses TIA	n (%) of readmissions	Readmission Type	(Year price) Reported cost (SD or IQR)	2021 US$ Cost PPP values**	Direct / Total (direct+indirect) % of total costs
Goeree, Canada, [46]	135	Not reported	All-cause	(2004)		
				Mean 12-month per patient		
				8095 CAD	9161	- / 46.0
Lee, Taiwan, [47]	686	290 (43.7)	All-cause	(2002)		
				Mean 12-month per patient		
				43157 NTD	3037	6.2 / - *
Luengo-Fernandez, England, [63]	295	Not reported	Cerebrovascular complications	(2009)		
		0.17 per patient/year	(within 7 days of subsequent vascular event)	Mean 12-month per index-hospitalization surviving patient		NC
				720 (3252) GBP	1273 (5751)	29.8 / - *
Porsdal, Denmark, [33]	73	5 (7.8)	Stroke-related	(1995)		
				Mean 12-month per index-hospitalization surviving patient		
				1650 DKK	360	8.8 / - *
Stowers, USA, [28]	985,851	91,261 (9.8) (30 day)	All-cause	(2014)		
		34,503 (3.7)*		Mean 7-day per index-hospitalization surviving patient		Not reported
			Year 2009	9322 USD	10,526	
			Year 2014	10961 USD	12,377	
		91,261 (9.8) (30 day		Mean 30-day per index-hospitalization surviving patient		

Year 2009	9323 USD	10,528
Year 2010	9754 USD	11,014
Year 2011	10,735 USD	12,122
Year 2012	10,336 USD	11,671
Year 2013	10,822 USD	12,220
Year 2014	11,320 USD	12,783
	Weighted mean per index-hospitalization surviving patient*	
	10,327 USD	11,661

TIA, Transient Ischemic Accident; USA, United States of America; CAD, Canadian Dollar; DKK, Danish Krona; GBP, Great Britain Pound; NTD, New Taiwanese Dollars; USD, US Dollar; PPP, Purchase Parity Prices; * Authors’ calculation based on articles data; **Purchase Parity Prices calculated with CCEMG – EPPI-Centre Cost Converter (https://eppi.ioe.ac.uk/costconversion/default.aspx)
