# Supplementary material for: Rapid systematic review of readmissions costs after stroke
Source: Cost Eff Resour Alloc. 2024 Mar 12;22:22. doi: 10.1186/s12962-024-00518-3 (PMC10936094; doi:10.1186/s12962-024-00518-3)
Supplement: Supplementary file 7 — Supplementary Material 7 [file 12962_2024_518_MOESM7_ESM.pdf]

Appendix Supplemental table 7 – Ischemic stroke readmission costs

Study characterization		Readmission characterization		Costs description		
Study, Country, [Ref.]	Sample size for economic analyses	n (%) of readmissions	Readmission Type	(Year price)<br>Reported cost (SD or IQR)	2021 US$ Cost PPP values	Direct / Total (direct+indirect)<br>% of total costs
Cadilhac, Australia, [55]	27,660	NR	Planned and unplanned	(2004)		
			Stroke-complications	mean 12-month per index-hospitalization surviving patient		
			IS/Und	1442 AUSD	1542	5.8 / -
			Stroke-recurrence	mean 12-month per index-hospitalization surviving patient		
			IS/Und	801 AUSD	857	3.2 / -
Caro, Canada, [37]	18,695	13,591 (72.7)<br>(per 5-years)	Planned and unplanned	(2002)		Not clear
			Cardiovascular disease	Total annual cost		
				24,000,000 CAD	28,959,889	
				Mean 12-month per index-hospitalization surviving patient *		
				1284 CAD	1549	-
			Bleeds	Total annual cost		
				5,000,000 CAD	6,033,310	
				Mean 12-month per index-hospitalization surviving patient *		
				267 CAD	322	-
Chang, Taiwan, [40]	1180 (TIA 686)	573 (48.6 of IS)	Planned and unplanned	(2002)		
			All-cause	Weighted mean 12-month per patient *		
				49,857 NTD	3523	29.1 / - *

Christensen, Scotland, [56]	4295	IS 1624 (44.8)	Planned and unplanned	(2006)		
			Stroke-recurrence/cardiovascular disease	Mean 12-month per patient		
				3127 (8101) GBP	5926 (15,353)	22.3 / -
				Mean 12-month per patient readmitted		
				8269 (11,449) GBP	15,672 (21,699)	-
				Mean 12-month per index-hospitalization surviving patient		
				3702 (8693) GBP	7016 (16,475)	-
				Mean 12-month per readmission		
				4487 (7669) GBP	8504 (14,535)	-
Gloede, Australia, [62]	243	NR	Unplanned	(2010)		
			Stroke-recurrence	Mean 12-month per readmission		
			IS	32,354 AUD	26,788	Not reported
			Stroke-complications	(3–5 years) 12-month mean per index-hospitalization surviving patient		
				117 USD	150	2.2 / -
				(10 years)12-month mean per index-hospitalization surviving patient		
				108 USD	132	2.1 / -
Goeree, Canada, [46]	188	NR	Planned and unplanned	(2004)		
			All-cause	Mean 12-month per patient		
				11,162 CAD	12,632	- / 21.0
Hellsten, Canada, [29]	29724 (30 days 28642, 90 days 27863)	2635 (9.2) 30 days[a] 5461 (19.6) 90 days*	Planned and unplanned	(2012)		
			All-cause	Mean (4 years) 30-day per patient surviving index-hospitalization		
				677 (451-872) CAD	642 (423-823)	2.8 / -

				Mean (4 years) 90-day mean per patient surviving index-hospitalization		
				2060 (1430-2924) CAD	1955 (1357-2775)	6.6 /-
				Mean (4 years) 30-day per readmission		
				8145 (5136-9107) CAD	7729 (4874-8642)	-
				Mean (4 years) 90-day per readmission		
				13068 (10308-16,142) CAD	12,401 (9782-15,318)	-
Johnson, USA, [30]	51251 (Medicare 31037)	12,042 (39.0) Medicare sample	Planned and unplanned	(2013)		
			Stroke-complications	30-day total (Medicare)*		
				144,504,000 USD	1,666,260,192	15.1 / - *
			Stroke-complications	Mean per 30-day readmission cost		
				12,000 USD	13,807	Not clear
Lee, USA, [35]	9131	5743 (62.9)* Medicare sample	Planned and unplanned	(2001)		
			All-cause	Mean per year (four-year) per patient*		
				11,895 USD	17,453	30.1 / - *
Lee, Taiwan, [47]	1180	482 (43.5)	Planned and unplanned	(2002)		
			All-cause	Mean 12-month per patient		
				53,541 NTD	3768	23.7 / - *
McGuire, Scotland, [36]	8893	5093 (57.2) per 11-years 20% per year	Planned and unplanned	(2005)		
			All-cause	Mean per year (eleven-years), per patient		
				10,262 (19,046) GBP	20,019 (37,155)	50.8 / - *
		1985	All-cause	Mean per index-hospitalization surviving patient (eleven years)		
				14,681 (21,307) GBP	28,640 (41,566)	54 / -

		24,302	All-cause	Mean readmission cost per readmission		
				3783 (8057) GBP	7380 (15,718)	-
Meretoja, Finland, [48]	8204 (year 2007)	Not reported	Planned and unplanned	(2008)		
			All-cause	Mean 12-month per patient – year 2007		
				6913 USD	6486	23.4 / - *
Porsdal, Denmark, [64]	340	56 (16)	Planned and unplanned	(1995)		
			Stroke-related	Mean 12-month per index-hospitalization surviving patient*		
				9380 DKK	2043	- / 20.0
Spieler, France, [65]	435	NR	Planned and unplanned	(1997)		
			All-cause	Total expenditure		
				254,144 EUR	446,459	-
				Mean 12-month per index-hospitalization surviving patient		
				584 (445-724) EUR	1010 (770-1252)	3.3 / - *
Spieler, France, [66]	435	NR	Planned and unplanned	(1997)		
			All-cause	Total 18-month expenditure		
			First-ever	227,779 EUR	397,692	-
			Recurrent	63,119 EUR	110,203	-
			All-cause	Mean 18-month per index-hospitalization surviving patient		
			First-ever	658 (479-837) EUR	1149 (836-1461)	3.3 / - *
			7274 (3.7)*	709 (413-1005) EUR	1238 (721-1755)	3.8 / - *
				Weighted mean per index-hospitalization surviving patient *		
				668 EUR	1166	3.4 / - *
Stein, USA, [67]	192,594	24,545 (12.7)*	Planned and unplanned	(2013)		

		All-cause	Mean 30-day per patient readmitted (unadjusted)		Not reported
	17,271 (8.9)*	Same hospital	51,087 (49,878-52,296) USD	52,054 (50,822-53,285)	-
	7274 (3.7)*	Different hospital	61,633 (not clear) USD	62,798	-
			Weighted mean per patient readmitted *		
			54,212 USD	55,238	-

TIA, Transient Ischemic Accident; IS, ischemic Stroke; USA- United Sates of America; USA- United Sates of America; AUSD, Australian Dollar; CAD, Canadian Dollar; DKK, Danish Krona; EUR, Euro; GBP, Great Britain Pound; NTD, New Taiwanese Dollars; PST, Pesetas; USD, US Dollar; SEK, Swedish Krona; Purchase Parity Prices: * Authors' calculation based on articles data; **Purchase Parity Prices calculated with CCEMG – EPPI-Centre Cost Converter (https://eppi.ioe.ac.uk/costconversion/default.aspx)
