# Supplementary material for: Rapid systematic review of readmissions costs after stroke
Source: Cost Eff Resour Alloc. 2024 Mar 12;22:22. doi: 10.1186/s12962-024-00518-3 (PMC10936094; doi:10.1186/s12962-024-00518-3)
Supplement: Supplementary file 8 — Supplementary Material 8 [file 12962_2024_518_MOESM8_ESM.pdf]

Appendix Supplemental Table 8 – Hemorrhagic stroke readmission costs

Study characterization		Readmission characterization		Costs description		
Study, Country, [Ref.]	Sample size for economic analyses	n (%) of readmissions	Readmission Type	(Year price) Readmission cost type Reported cost (SD or IQR)	2021 US$ Cost PPP values**	Direct / Total (direct+indirect) % of total costs
Cadilhac, Australia, [55]	4291	Not reported	Planned and unplanned	(2004)		
			Stroke-complications	Mean 12-month per index-hospitalization surviving patient		
				482 AUSD	516	1.6 / -
			Stroke-recurrence			
				460 AUSD	492	1.6 / -
Chang, Taiwan, [40]	2368 HS (HS: ICH 424, SAH 78)	ICH 267 (11.3) SAH 46 (19.4) (death/readmission)	Planned and unplanned	(2002)	(2002)	
			All-cause (HS)	Weighted mean per patient*		
				51,514 NTD	3640	22.9 / - *
Christensen, Scotland, [56]	1016	243 (43.6)	Planned and unplanned	(2006)		
			Stroke-recurrence/cardiovascular disease	Mean 12-month per patient		
				1918 (7248) GBP	3635 (13,737)	13.7 / - *
				Mean 12-month per patient readmitted		
				8011 (13,089) GBP	15,183 (26,171)	-
				Mean 12-month per index-hospitalization surviving patient		
				3495 (9507) GBP	6624 (18,018)	-
				Mean 12-month per readmission		

				4022 (8442) GBP	7623 (15,600)	-
Gloede, Australia, [62]	43	Not reported	Planned and unplanned	(2010)		
			Stroke-recurrence	Mean 12-month per readmission (first-year)		
				32,354 AUD	26,788	Not reported
			Stroke-complications	(3–5 years) 12-month mean per index-hospitalization surviving patient		
				183 USD	223	3.1 / -
				(10 years)12-month mean per index-hospitalization surviving patient		
				26 USD	32	0.3 / -
Goeree, Canada, [46]	42	Not reported	Planned and unplanned	(2004)		
			All-cause	Mean 12-month per patient		
				5218 CAD	5905	- / 9.0
Hellsten, Canada, [29]	7304<br>(30 days 7014; 90 days 6845)	554 (7,9) 30 days*<br>1061 (15,5) 90days*	Planned and unplanned	(2012)		
			All-cause	Mean (4 years) 30-day per patient surving index hospitalization		
				715 (365-1410) CAD	679 (679-1338)	2.8 / -
			All-cause	Mean per (4 years) 90-day per patient surving index hospitalization		
				1898 (1430-2924) CAD	1801 (1357-2775)	6.1 / -
				Mean per (4 years) 30-day per readmission		
				10,035 (6208-13,884) CAD	9523 (5891-13,175)	
				Mean per (4 years) 90-day per readmission		
				15,524 (9656-20,839) CAD	14,732 (9163-19,775)	
Hoffmann, USA, [68]	64,609	8372 (12.9)<br>30 days rate/5 years	Planned and unplanned	(2019)		Not reported
			All-cause	Five-year total readmission cost		
				160,320,059 USD	167,069,927	
				Median 30-day cost of individual readmissions		

				10,342 (5641-19,584) USD	10,777 (5879-20,409)	
Lee, USA, [35]	1757	1009* (60.9)	Planned and unplanned	(2001)		
			All-cause	Mean per year (four-year) per patient*		
				11,601 USD	17,022	30.5 / - *
Lee, Taiwan, [47]	424	142 (44.7)	Planned and unplanned	(2002)		
			All-cause	Mean 12-month per patient		
				58,278 NTD	4102	35.6 / - *
McGuire, Scotland, [36]	705	308 (43,7) per 11-years	Planned and unplanned	(2005)		
			All-cause	Mean per year (eleven-years), per patient		
				7252 (18,396) GBP	14,147 (35,887)	44.5 / - *
				Mean readmission cost per index-hospitalization surviving patient		
				13,349 (23,382) GBP	26,041 (45,614)	45 / -
				Mean readmission cost per readmission		
				3240 (7349) GBP	6321 (14,336)	
Meretoja, Finland, [48]	1413<br>(year 2007)	Not reported	Planned and unplanned	(2008)		
			All-cause	Mean 12-month per patient – year 2007		
				4703 USD	3821	13 / - *

ICH, Intracerebral Hemorrhage; SAH, Subarachnoid Hemorrhage; USA, United Sates of America; AUSD, Australian Dollar; CAD, Canadian Dollar; DKK, GBP, Great Britain Pound; NTD, New Taiwanese Dollars; PST, Pesetas; USD, US Dollar; PPP, Purchase Parity Prices; * Authors' calculation based on articles data; **Purchase Parity Prices calculated with CCEMG – EPPI-Centre Cost Converter (https://eppi.ioe.ac.uk/costconversion/default.aspx)
