# Supplementary material for: Rapid systematic review of readmissions costs after stroke
Source: Cost Eff Resour Alloc. 2024 Mar 12;22:22. doi: 10.1186/s12962-024-00518-3 (PMC10936094; doi:10.1186/s12962-024-00518-3)
Supplement: Supplementary file 9 — Supplementary Material 9 [file 12962_2024_518_MOESM9_ESM.pdf]

Appendix Supplemental Table 9 – Subarachnoid haemorrhage readmission costs

Study characterization		Readmission characterization		Costs description		
Study, Country, [Ref.]	Sample size for economic analyses	n (%) of readmissions	Readmission Type	(Year price) Readmission cost type Reported cost (SD or IQR)	2021 US$ Cost PPP values	Direct / Total (direct+indirect) % of total costs
Lee, USA, [35]	342	184* (53.6)	Planned and unplanned	(2001)		
			All-cause	Mean per year (four-years) per patient		
				14, 545 USD	21,342	30.1 / - *
Lee, Taiwan, [47]	78	9 (22.0)	Planned and unplanned	(2002)		
			All-cause	Mean 12-month per patient		
				13,423 NTD	945	31.9 / - *
Meretoja, Finland, [48]	721	Not reported	Planned and unplanned	(2008)		
			All-cause	Mean 12-month per patient – year 2007		
				5339 USD	5009	12.5 / - *

SAH, Subarachnoid Haemorrhage; USA, United Sates of America; CAD, Canadian Dollar; DKK, Danish Krona; GBP, Great Britain Pound; NTD, New Taiwanese Dollars; USD, US Dollar; PPP, Purchase Parity Prices; * Authors' calculation based on articles data; **Purchase Parity Prices calculated with CCEMG – EPPI-Centre Cost Converter (https://eppi.ioe.ac.uk/costconversion/default.aspx)
